# Knockdown of p53 Enhances LncRNA A2M‐AS1 Inhibition of Pancreatic Cancer Progression via Regulating MAPK Pathway

**DOI:** 10.1002/cam4.70956

**Published:** 2025-07-13

**Authors:** Yu Cen, Yihui Luo, Yi Nan, Wenxi Kuang, Xianglian Zhang, Jiexia Lu, Chunxiao Xie, Mingzhi Xie, Enran Chen, Haixing Jiang, Shanyu Qin

**Affiliations:** ^1^ Department of Gastroenterology The First Affiliated Hospital of Guangxi Medical University Nanning China; ^2^ Department of Gastroenterology Liuzhou People's Hospital Liuzhou China; ^3^ Department of Gastroenterology The People's Hospital of Guangxi Zhuang Autonomous Region Nanning Guangxi Province China; ^4^ Department of General Medicine Guangxi Medical University Nanning China

**Keywords:** LncRNA A2M‐AS1, p53, pancreatic cancer, therapeutic target

## Abstract

**Purpose:**

Rapid progression in late‐stage is a characteristic of pancreatic cancer (PC), leading to mortality. The critical role of lncRNA A2M‐AS1 (long non‐coding RNA alpha‐2‐macroglobulin antisense RNA 1) is involved in cancer progression, but the upstream regulator of A2M‐AS1 in the PC progression phenotype remains elusive.

**Methods:**

We conducted an integrated analysis using bioinformatics, in vitro experiments, and in vivo studies. Human PC tissues were analyzed for A2M‐AS1 and p53 expressions. The PANC‐1 and BxPC‐3 cell lines were used for functional assays, including cell proliferation, apoptosis, migration, and invasion assays. The role of p53 in regulating A2M‐AS1 was investigated through overexpression and knockdown studies, along with using a MAPK pathway inhibitor.

**Results:**

We found that A2M‐AS1 is downregulated in PC tissues and that its high expression correlates with a better prognosis. p53 was identified as a negative regulator of A2M‐AS1, with its knockdown leading to increased A2M‐AS1 expression and decreased PC cell invasiveness. Mechanistically, p53 was shown to bind to the A2M‐AS1 promoter, modulating its transcriptional activity. The MAPK pathway was revealed as a downstream effector of the p53‐A2M‐AS1 axis, with its inhibition reversing the effects on PC cell behavior.

**Conclusion:**

High A2M‐AS1 expression is associated with a better PC prognosis. A2M‐AS1 overexpression subdues PC cell development. Our study unveils a new mechanism by which p53 decreases A2M‐AS1 expression.

## Graphical abstract

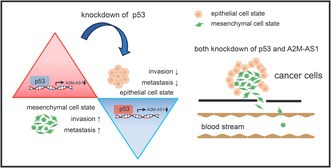

**Abbreviations**
FISH: fluorescence in situ hybridization
IHC: immunohistochemistry
LncRNA A2M‐AS1: long non‐coding RNA alpha‐2‐macroglobulin antisense RNA 1
PC: pancreatic cancer/pancreatic carcinoma
TCGA: The Cancer Genome Atlas

## 1 Introduction

Pancreatic carcinoma (PC) is a prevalent malignant tumor of the digestive tract with a 5‐year survival rate of < 10% and is projected to become the second leading cause of cancer‐related deaths by 2030 [1]. A high rate of metastasis in the early stage is the main cause of PC‐related mortality. In addition, the lack of effective screening methods and non‐specific symptoms results in late‐stage diagnosis when surgical resection is difficult or impossible [2].

Revealing the molecular mechanisms governing the invasion and metastasis of PC cells is imperative for enhancing therapeutic outcomes. A2M‐AS1 is an antisense chain of alpha‐2‐macroglobulin (A2M) genes with a sequence complementary to A2M. A2M‐AS1 is involved in tumor development, including breast cancer, lung cancer, pancreatic cancer, and gastric cancer, among others [3]. Our research group revealed that A2M‐AS1 has a good diagnostic and prognostic value for PC and is mainly located in the cytoplasm of PC cells. A2M‐AS1 overexpression suppresses tumor progression and metastasis [4]. The A2M‐AS1 upstream regulator has not been reported, and its accurate underlying molecular mechanism remains elusive.

Therefore, this study aimed to investigate the precise role of A2M‐AS1 and its upstream regulator in PC. A2M‐AS1 displayed a low expression in human PC tissues compared with adjacent normal pancreatic tissues, inhibiting the PC cell metastatic phenotype in vitro. Subsequently, we aimed to identify upstream regulators of A2M‐AS1 by bioinformatics analysis, identifying STAT1 and p53 as target genes. Unexpectedly, IFN‐γ, which is an agonist of STAT1, could not change the tumor progression and metastatic phenotype of PC cells. For further analysis, we concentrated on the *TP*53 gene, which was upregulated in PC tissues and inhibited A2M‐AS1 in PC cells. *TP*53 encodes the tumor suppressor protein p53, which acts as a sequence‐specific transcription factor binding to specific DNA sequences in the genome [5]. According to previous reports, a variety of signal pathways (such as MAPK, NF‐κB, Wnt/β‐Catenin, and STAT3) are downstream effective targets [6, 7, 8]. After enrichment analysis, we demonstrated that p53‐A2M‐AS1 affected the MAPK pathway in PC, which could reverse PC suppression caused by p53‐A2M‐AS1. Therefore, these findings suggest a novel explanation of the mechanisms involved in p53‐A2M‐AS1‐mediated PC metastasis.

## 2 Materials and Methods

### 2.1 Reagents and Antibodies

The PANC‐1 and BxPC‐3 cell lines were purchased from the Chinese Academy of Sciences (Shanghai, China) cell bank. IFN‐γ and fludarabine were obtained from R&D Systems (Minneapolis, MN). The MAPK inhibitor (PD184352) was obtained from Cell Signaling Technology (Beverly, MA, USA). Antibodies to p53 and glyceraldehyde 3‐phosphate dehydrogenase (GAPDH) were purchased from ABclonal Technology (Wuhan, China). Antibodies to p‐JNK, p‐MEK, p‐Raf, P44, p‐cJUN, and Ras were purchased from Cell Signaling Technology (Beverly, MA, USA). A2M‐AS1 overexpression lentivirus and siRNA were constructed by the Gene Chem Company (Shanghai, China).

### 2.2 Patients and Samples

A total of 32 paired tumor tissues and matched adjacent tissues (3 cm away from the tumor edge) were surgically resected from patients with PC treated at The First Affiliated Hospital of Guangxi Medical University between January 2017 and December 2022. The patients were pathologically diagnosed with PC and received no preoperative antitumor therapy. The basic clinicopathological data of the patients are provided in Table S1. The Ethics Committee of The First Affiliated Hospital of Guangxi Medical University approved this study. Written informed consent was waived by the ethics committee because this study was fundamental and used supplementary tumor tissues left after diagnostic workup and because all data were anonymized.

### 2.3 Cell Culture

PANC‐1 and BxPC‐3 cells were respectively cultured in Dulbecco's Modified Eagle Medium (DMEM) or Roswell Park Memorial Institute (RPMI) 1640 medium supplemented with 10% fetal bovine serum (FBS) (Gibco, NY, USA), 100 U/mL penicillin (Meilunbio, Dalian, China), and 100 μg/mL streptomycin (Meilunbio, Dalian, China) in a humidified atmosphere containing 5% CO_2_/95% air at 37°C.

In order to determine the effect of IFN‐γ, PC cells were directly added to a normally cultured group, followed by incubation for the time indicated in the figure legends. In order to determine the effect of STAT1 inhibitor (fludarabine) inhibition on IFN‐γ‐induced ferroptosis, PC cells were incubated with fludarabine (13.5 μM) for 48 h in the presence or absence of 200 ng/mL IFN‐γ.

In order to examine the A2M‐AS1 effect on PC cells, PANC‐1 cells were cultured with overexpressed A2M‐AS1 lentivirus, and BxPC‐3 cells were transfected with A2M‐AS1 siRNA for 12 h. In order to clarify the effect of *TP*53 inhibition on A2M‐AS1 in PC cells, PANC‐1 cells were cultured with *TP*53 siRNA based on the overexpression or knockdown of A2M‐AS1. In order to obtain stable cell lines, transfected cells were selected for 2 weeks by puromycin (2 μg/mL). Lipofectamine 2000 was used for transient transfection, following the manufacturer's instructions. The cells were saved for subsequent cell assays 48 h after transfection.

### 2.4 Cell Proliferation Assays

Cellular proliferation was assessed by the Cell Counting Kit‐8 (CCK‐8; Meilunbio, Dalian, China). Briefly, transfected cells were inoculated in 96‐well plates at 5000 cells/well and cultured for 0–6 days. A medium containing 10% CCK‐8 was added to each well at each time point. All samples were incubated at 37°C for 2 h, and a microplate reader (Nikon, Tokyo, Japan) was used to evaluate the absorbance at 450 nm.

### 2.5 Apoptosis Assay

Apoptosis was evaluated using an Annexin V‐7AAD Apoptosis Kit (BD, USA). The cells were resuspended into a binding buffer (BD, USA), after which 100 × 10^4^ cells/mL (100 μL) were transferred into FACS tubes, stained with 5 μL of Annexin 7AAD for 20 min and 5 μL of PI for 5 min. A flow cytometer system (BD, USA) was used to determine the apoptosis rates of each group within 30 min.

### 2.6 Migration and Invasion Assay

The stably transfected cells were inoculated in 6‐well plates (10^6^ cells/well) and incubated overnight for the migration assay. Scratches were made using a 10 μL tip perpendicular to the plate bottom and photographed under the microscope to calculate the wound healing after 0 and 72 h.

For the invasion assay, the cells were added to a 200 μL serum‐free medium at 10^5^ cells/well and placed in the upper chamber of a Transwell system (the Matrigel was laid 2 h before). A total of 500 μL complete medium containing 10% FBS was added into the lower chamber. After incubation for 72 h, the cells on the lower side of the chamber were fixed, stained, and photographed under a microscope. Three visual fields in each chamber were randomly selected for further enumeration.

### 2.7 Fluorescence In Situ Hybridization (FISH)

The expression of A2M‐AS1 in 32 PC specimens was examined using a lncRNA FISH kit (GenePharma, Shanghai, China). The positive control group was established using 18S‐biotin and the target genome A2M‐AS1, while the negative control group used NC‐Biotin. Paraffin‐embedded pathological sections were dewaxed in xylene. The sections were rehydrated through graded alcohols, fixed, and treated with 100 μL proteinase K for 20 min at 37°C. The sections were incubated for 8 min at 78°C and incubated with 100 μL of probe‐containing hybridization solution at 37°C overnight. The sections were incubated with a scrubbing solution for 15 min at 37°C. The hybridized cells were counterstained with DAPI and observed under a fluorescence microscope.

### 2.8 RNA Isolation and qRT‐PCR Assays

TRIzol reagent (Invitrogen, USA) was used for RNA extraction. PrimeScript RT‐PCR Kit (TaKaRa, Haidian, Beijing, China) was used for reverse transcription. A nucleic acid level evaluation was performed using SYBR Green qPCR Master Mix (TaKaRa, Haidian, Beijing, China). The ABI 7500 Real‐Time PCR System (Biosystems, USA) was used. The adjustment of reaction conditions followed the users' instructions. The GAPDH was used to standardize A2M‐AS1 expression, and the 2^−ΔΔCt^ method was applied to quantify the relative gene expression. The primer sequences utilized in this investigation are documented in Table 1.

**TABLE 1:** The primer sequences.

Gene	Sequence
A2M‐AS1‐F	GCAGCAGCTTGTGTGTGTGA
A2M‐AS1‐R	GGAGATTGAGACGGGTGGAG
TP53‐F	ACAGCTTTGAGGTGCGTGTTT
TP53‐R	CCCTTTCTTGCGGAGATTCTCT
GAPDH	GCACCGTCAAGGCTGAGAAC
GAPDH	TGGTGAAGACGCCAGTGGA

### 2.9 Immunohistochemistry (IHC)

The expression of p53 was assessed using IHC in 38 patients diagnosed with PC. The samples comprised various pathological types, including pancreatic ductal adenocarcinoma, adenosquamous, and glial carcinoma. After excluding samples with fragmented sections or incomplete information, 32 specimens were included in this study. Paraffin‐embedded tissues were sectioned at a thickness of 4 μm. Antigen retrieval was performed using a pressure cooker for 3 min in 0.01 M citrate buffer (pH 6.0). The sections were incubated overnight at 4°C with antibodies specific for p53 at a dilution of 1:50. Immunodetection was conducted the following day using DAB. Tissue staining was observed by microscope, after which the images were collected. The Image Pro Plus software was applied to analyze the IHC images. The images were converted into 8‐bit grayscale images, the positive threshold was set, and the grayscale value (IOD value) and positive area size (area) were determined. The average optical density (AOD) was calculated using the formula AOD = IOD/area. According to the average AOD value of IHC, the patients were divided into the high‐ and low‐expression groups.

### 2.10 Immunoblot Analyses

Total proteins were extracted with RIPA lysis buffer (Solarbio, Beijing, China) and centrifuged. The supernatants were collected for densitometry analysis. Proteins (25 mg) were separated by SDS‐PAGE and transferred to polyvinylidene fluoride membranes. After incubation with 5% nonfat milk for 1 h at room temperature, the membranes were incubated with a primary antibody overnight at 4°C and then with an appropriate horseradish peroxidase‐conjugated secondary antibody for 1 h at room temperature. Bands were detected using an ECL reagent (Affinity Bio, Victoria, Australia). The intensity of the immunoblot results was determined by Image J Software (version 1.52 k, NIH).

### 2.11 Luciferase Reporter Assays

The fragment sequences were synthesized and inserted into a pGL3‐basic vector. Before transfection, 1 × 10^5^ 293 T cells were inoculated in 48‐well plates with 300 μL DMEM complete medium per well without antibiotics and cultured overnight in cell incubators. Transfection was performed at 70%–80% confluence. The plasmid combination (167 μg per plasmid, a total of 500 μg plasmid) was diluted in 25 μL of serum‐free high‐glucose DMEM medium. Before use, 1.5 μL of EZ Cell Transfection Reagent was diluted into 25 μL of serum‐free high‐glucose DMEM medium for each well. Diluted EZ Trans transfection reagent was added to the diluted plasmid DNA and incubated at room temperature for 10–15 min. A total of 300 μL medium transfection complex was added to the well plate. After culturing for 24 h, the medium containing the transfection complex was discarded and replaced with DMEM complete medium. After 24 h of culture, the medium was refreshed.

### 2.12 In Vivo Experiment

Ethical approval for all animal experiments was obtained from the Animal Ethics Committee of Guangxi Medical University (Nanning, China). Male nude mice (3–4 weeks old; 14–18 g) were randomly allocated into two experimental groups: the sh‐TP53 + OE‐A2M‐AS1 group and the sh‐TP53 + sh‐A2M‐AS1 group. Each group received intraperitoneal injections of cell suspensions containing 1 × 10^6^ PC cells in 1 mL phosphate‐buffered saline (PBS). After 1 month, the mice were humanely euthanized to evaluate tumor size and weight.

### 2.13 Statistical Analysis

SPSS 25.0 (IL, USA) was used for all statistical analyses. Differences between the two groups were analyzed using the *t*‐test and the chi‐squared test. Differences between multiple groups were analyzed using one‐ and multi‐way ANOVA. The survival time and influence of p53 and A2M‐AS1 expression on the prognosis were analyzed using the Kaplan–Meier method and the log‐rank test.

## 3 Results

### 3.1 A2M‐AS1 Has a Low Expression in Human PC Specimens and Is Associated With a Better Prognosis

**FIGURE 1:**
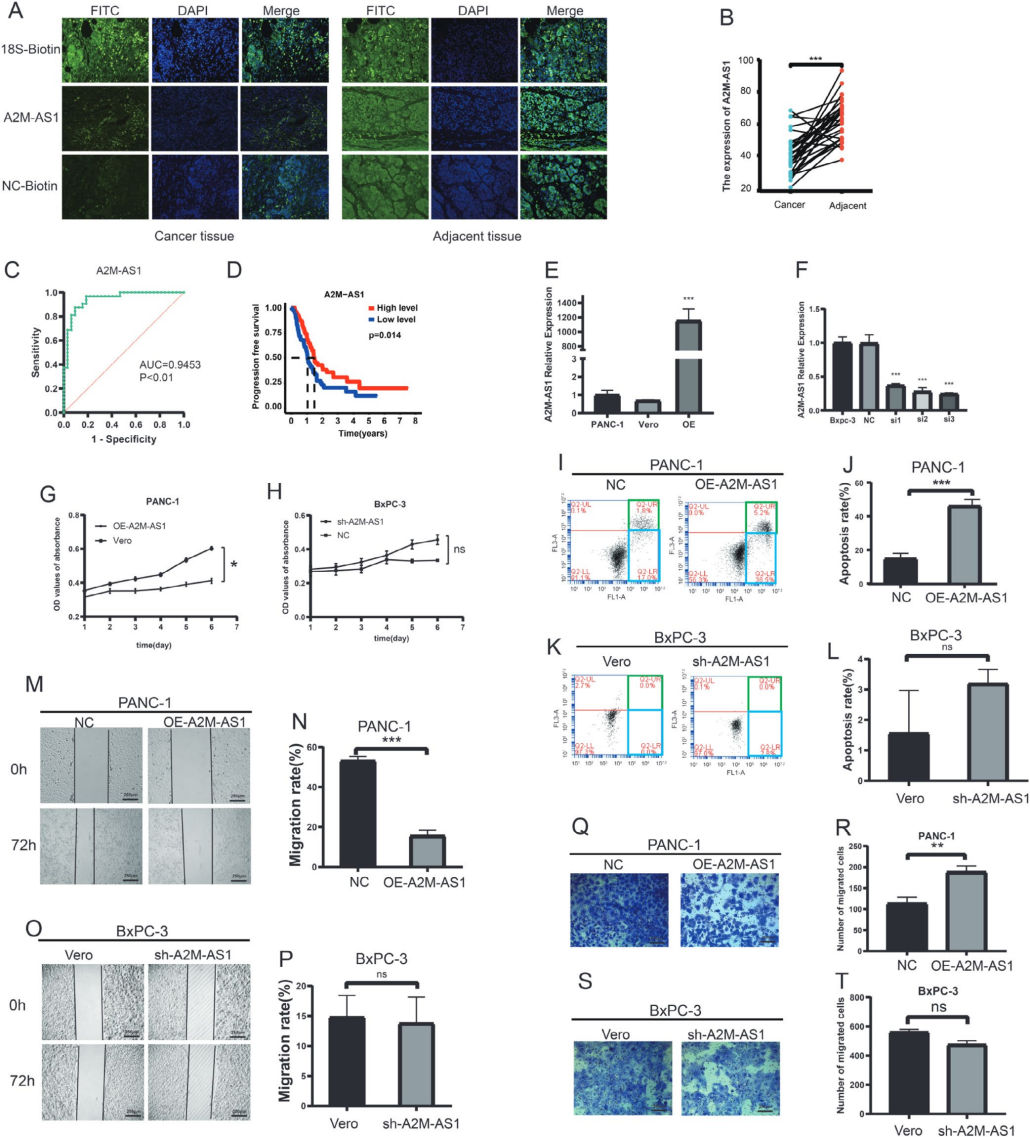
A2M‐AS1 expression is downregulated in PC tissues and correlates with better outcomes in PC patients. (A) Representative image of Fluorescence in situ hybridization for A2M‐AS1 expression levels in PC tissue. Scale bar, 200 μm. (B) Comparison of A2M‐AS1 expression levels in PC tissues and the corresponding adjacent noncancerous tissues. Two‐tailed *t*‐test. ***p < 0.001. (C) ROC analysis to evaluate the predictive value of A2M‐AS1 levels for patient survival time. (D) Kaplan–Meier curve for overall survival by A2M‐AS1 levels according to A2M‐AS1 expression levels. (E) Overexpression of A2M‐AS1 (OE‐A2M‐AS1) in PANC‐1 as determined by qRT–PCR. ***p < 0.001. Vero is the empty virus control group of sh‐A2M‐AS1. (F) Knockdown of A2M‐AS1 by three independent shRNAs in BxPC‐3 cells as determined by qRT–PCR. ***p < 0.001. (G, H) The effect of A2M‐AS1 overexpression on PANC‐1 and knockdown on BxPC‐3 proliferation. ns > 0.05. (I, J) Apoptosis assay following A2M‐AS1 overexpression in PANC‐1. ***p < 0.001. (K, L) Apoptosis assay following A2M‐AS1 knockdown in BxPC‐3. ***p < 0.001. (M, N) Scratch assay following A2M‐AS1 overexpression in PANC‐1. Scale bars, 200 μm.***p < 0.001. (O, P) Scratch assay following A2M‐AS1 knockdown in BxPC‐3. Scale bars, 200 μm. ns > 0.05. (Q, R) The effect of A2M‐AS1 overexpression on PANC‐1 invasion. Scale bars, 200 μm. **p < 0.01. (S, T) The effect of A2M‐AS1 knockdown on BxPC‐3 invasion. Scale bars, 200 μm. ns > 0.05.

In order to assess the clinical significance of A2M‐AS1 in PC, we conducted FISH staining on specimens from 38 patients with PC, including 32 pairs of primary tumor tissues and their corresponding adjacent noncancerous tissues. The findings revealed abundant expression of A2M‐AS1 in PC tissues (Figure 1A), with significantly higher levels observed in PC tissues compared with the adjacent tissue (Figure 1B). Receiver operating characteristic (ROC) curve analysis demonstrated that A2M‐AS1 could serve as a reliable prognostic indicator for PC (Figure 1C). Furthermore, survival analysis based on The Cancer Genome Atlas (TCGA) data showed that patients classified as “A2M‐AS1‐low” exhibited significantly shorter overall survival than those categorized as “A2M‐AS1‐high” (Figure 1D).

In our previous study, we observed that BxPC‐3 cells exhibited the highest expression level of A2M‐AS1, whereas PANC‐1 cells displayed the lowest expression level. In order to investigate the functional role of A2M‐AS1 in PC, we stably overexpressed A2M‐AS1 in PANC‐1 cells with low levels via lentiviral infection and stably transduced BxPC‐3 cells with lentiviral shRNA constructs targeting A2M‐AS1, as confirmed by qRT‐PCR analysis (Figure 1E,F). Notably, proliferation assays revealed ectopic overexpression of A2M‐AS1 induced PC cell proliferation (Figure 1G), while there was no significant impact on the knockdown of A2M‐AS1 (Figure 1H). However, flow cytometric analysis demonstrated that overexpression of A2M‐AS1 induced apoptosis in PANC‐1 cells (Figure 1I,J), while the knockdown of A2M‐A21 did not affect apoptosis in BxPC‐3 cells (Figure 1K,L). Consequently, our focus shifted toward investigating the potential role of A2M‐A21 in PC metastasis. Transwell and wound healing assays unveiled that ectopic expression of A2M‐A21 inhibited cell invasion and migration capabilities (Figure 1M,N,Q,R), whereas the knockdown of A2M‐AS1 did not promote cell migration and invasion abilities (Figure 1O,P,S,T).

### 3.2 IFN‐γ Influences PC Cell Apoptosis in an A2M‐AS1‐Independent Manner

**FIGURE 2:**
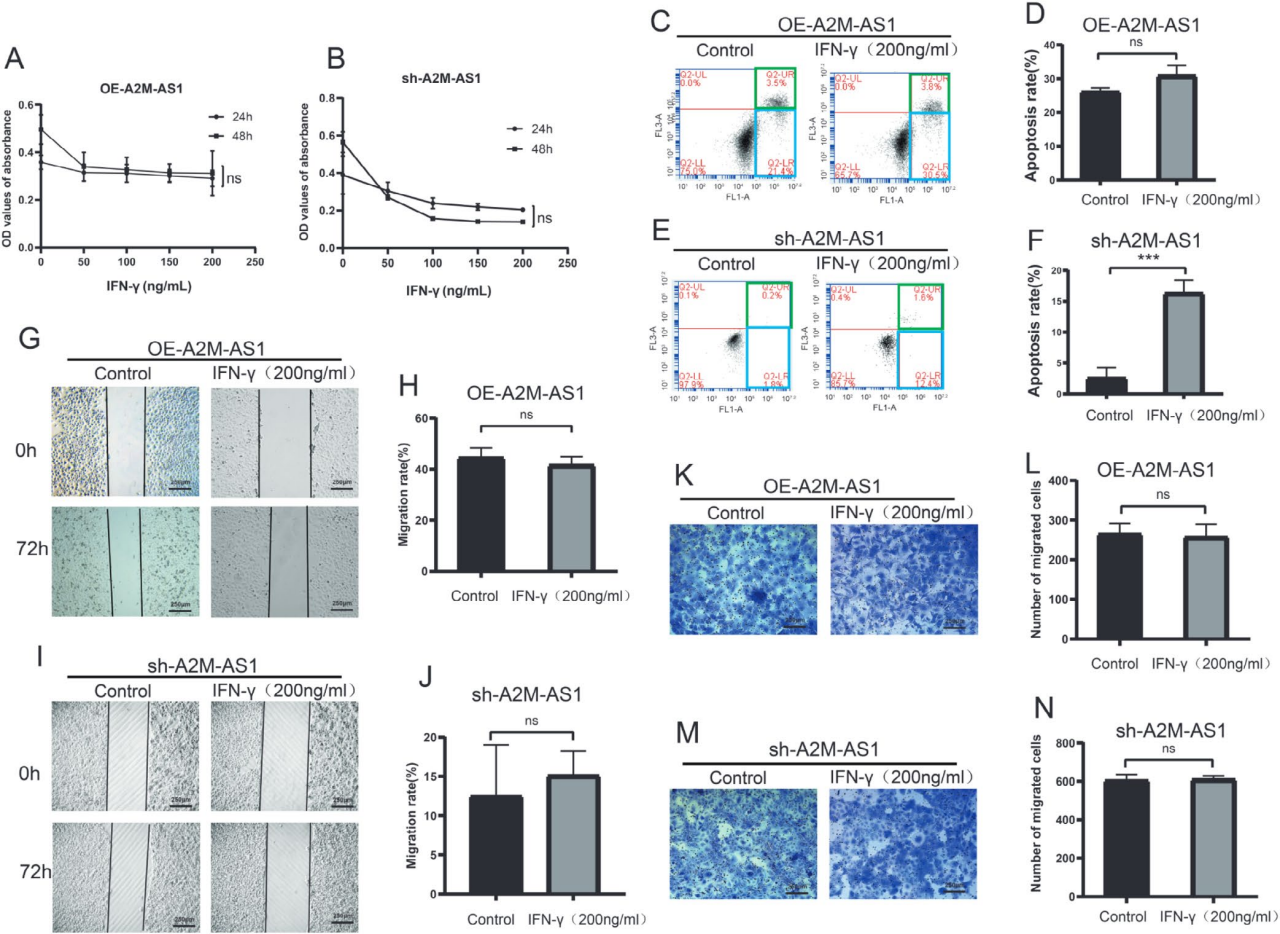
IFN‐γ influences PC cell migration and invasion in an A2M‐AS1‐independent manner. (A, B) The effect of IFN‐γ on A2M‐AS1 overexpression and A2M‐AS1 knockdown proliferation. ns > 0.05. (C, D) Apoptosis assay following IFN‐γ co‐culture with A2M‐AS1 overexpression in PANC‐1. ns > 0.05. (E, F) Apoptosis assay following IFN‐γ co‐culture with A2M‐AS1 knockdown in BxPC‐3. ***p < 0.001. (G, H) Scratch assay following IFN‐γ co‐culture with A2M‐AS1 overexpression in PANC‐1. Scale bars, 200 μm. ns > 0.05. (I, J) Scratch assay following IFN‐γ co‐culture with A2M‐AS1 knockdown in BxPC‐3. Scale bars, 200 μm. ns > 0.05. (K, L) The effect of IFN‐γ co‐culture with A2M‐AS1 overexpression on PANC‐1 invasion. Scale bars, 200 μm. ns > 0.05. (M, N) The effect of IFN‐γ co‐culture with A2M‐AS1 knockdown on BxPC‐3 invasion. Scale bars, 200 μm. ns > 0.05.

We initially co‐cultured PC cells with IFN‐γ, a STAT1 agonist. Proliferation assays revealed that IFN‐γ had no significant effect on the proliferation of PC cells, regardless of ectopic expression or knockdown of A2M‐AS1 (Figure 2A,B). Flow cytometric analysis demonstrated that IFN‐γ only induced apoptosis elevation in BxPC‐3 cells (Figure 2C–F). Transwell and wound healing assays indicated that IFN‐γ did not affect cell invasion and migration (Figure 2G–N). These findings suggest that the impact of IFN‐γ on PC cells does not occur through the A2M‐AS1 pathway.

### 3.3 p53 Is Negatively Associated With A2M‐AS1 Expression in PC

**FIGURE 3:**
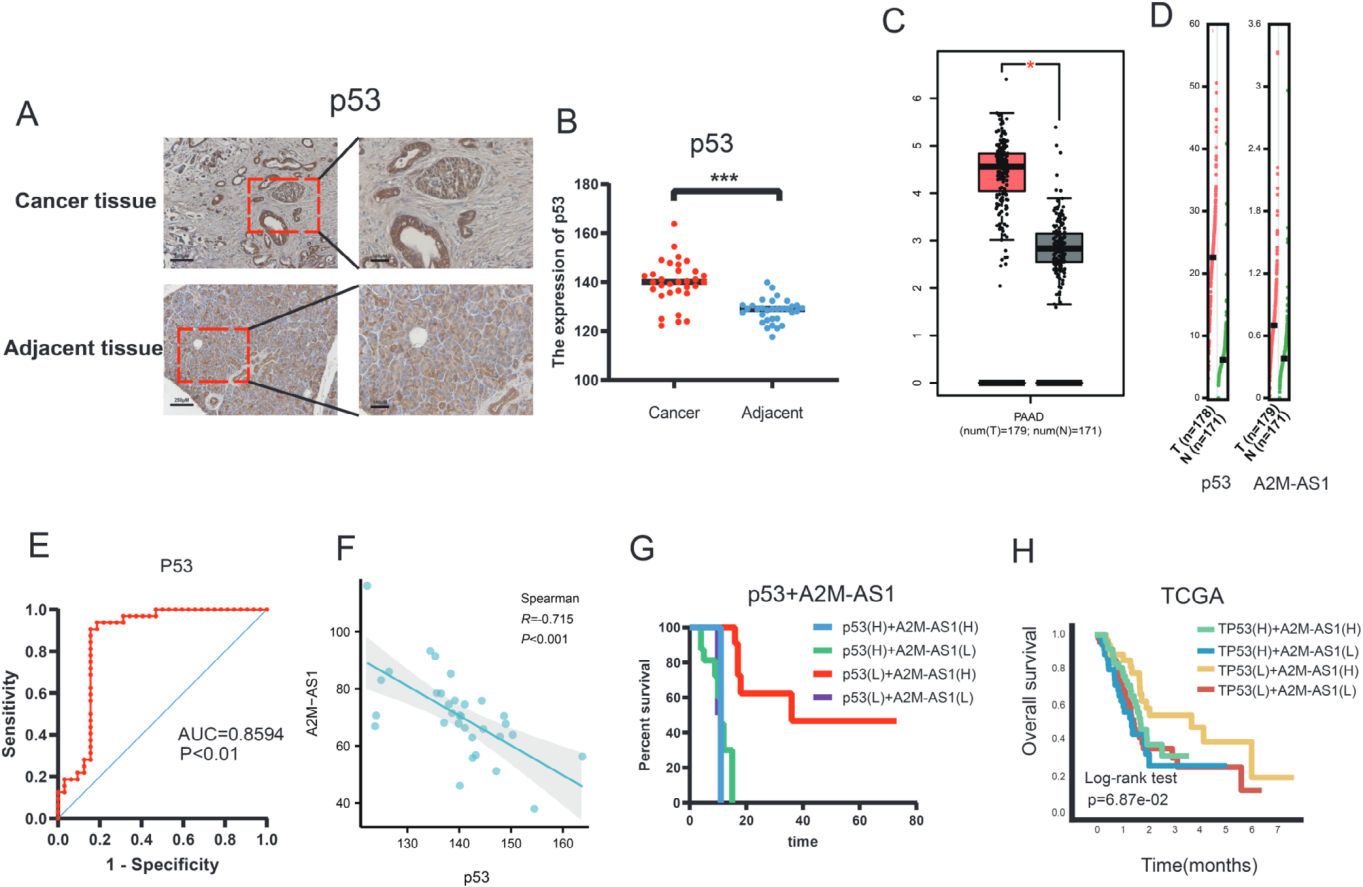
p53 expression level is negatively associated with A2M‐AS1 expression in PC. (A) Representative IHC staining of p53 in PC tissues. Scale bar, left, 200 μm; (B) Comparison of p53 staining scores in unpaired PC (tumor, *n* = 32) and adjacent noncancerous tissues (Adj NT, *n* = 32). Two‐tailed *t*‐test. ***p < 0.001. (C) p53 expression in TCGA PC and normal tissues. Two‐tailed *t*‐test. *p < 0. 05. (D) p53 and A2M‐AS1 expression in TCGA PC and normal tissues. (E) ROC analysis to evaluate the predictive value of p53 for patient survival time. (F) Correlations between p53 and A2M‐AS1. *R*, Pearson correlation coefficient. (G) Combined analysis of p53 and A2M‐AS1 expression levels in the prognostic value of patients with PC by Kaplan–Meier survival curves. (H) Survival plot in TCGA PC patients according to the combination of p53 and A2M‐AS1 expression.

In order to evaluate the clinical significance of p53 and A2M‐AS1 in PC, we compared their expression levels between normal and PC tissues using data from TCGA. We assessed the expression of p53 and A2M‐AS1 in 38 patients with PC. After excluding samples with fragmented sections or incomplete information, 32 patients were included for analysis. IHC demonstrated abundant protein expression of p53 in PC tissues (Figure 3A,B). Subsequently, our analysis revealed a significant upregulation of p53 in PC tissues compared with normal tissues. However, there were no notable differences in A2M‐AS1 expression between PC and normal tissues according to the TCGA database (Figure 3C,D). In addition, FISH was employed to examine the levels of A2M‐AS1 in PC samples. The ROC curves indicated that p53 could serve as prognostic markers for pancreatic cancer patients (Figure 3E). Importantly, our analysis revealed a strong negative correlation between the expression levels of p53 and A2M‐AS1 within TCGA datasets for patients with PC (Figure 3F). Furthermore, individuals with concurrent low levels of p53 and high levels of A2M‐AS1 exhibited significantly longer survival times among the 32 patients with PC studied here as well as within the TCGA datasets (Figure 3G,H). These findings strongly support the clinical relevance of both p53 and A2M‐AS1 in PC.

### 3.4 p53 Negatively Regulates A2M‐AS1 Expression

**FIGURE 4:**
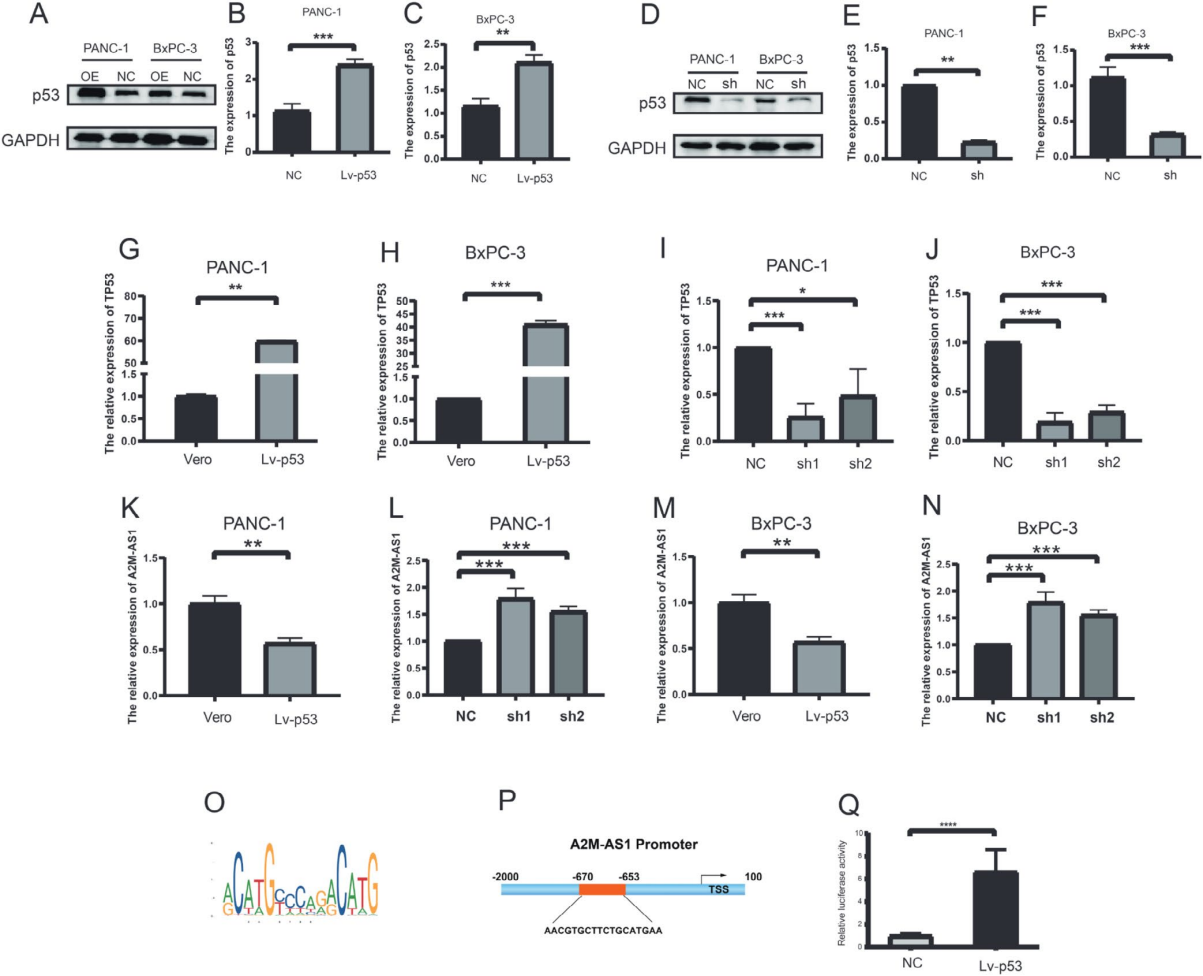
p53 negatively regulates A2M‐AS1 expression. (A–C) Overexpression of p53 (Lv‐p53) in PANC‐1 and BxPC‐3 cells as determined by Western blot. **p < 0.01, ***p < 0.001. (D–F) Knockdown of p53 by two independent shRNAs in PANC‐1 and BxPC‐3 cells as determined by Western blot. **p < 0.01, ***p < 0.001. (G, H) Overexpression of p53 (Lv‐p53) in PANC‐1 and BxPC‐3 cells as determined by qRT–PCR. **p < 0.01, ***p < 0.001. (I, J) Knockdown of p53 by two independent shRNAs in PANC‐1 and BxPC‐3 cells as determined by Western blot. *p < 0.05, ***p < 0.001. (K, L) qRT–PCR analysis of A2M‐AS1 expression with overexpression or knockdown of p53 in PANC‐1 cells. **p < 0.01, ***p < 0.001. (M, N) qRT–PCR analysis of A2M‐AS1 expression with overexpression or knockdown of p53 in BxPC‐3 cells. **p < 0.01, ***p < 0.001. (O) Promoter sequence of A2M‐AS1 predicted by JASPAR website. (P) p53 binding sites in A2M‐AS1 promoter sequence. (Q) Luciferase assay assessing A2M‐AS1 promoter activity, with p53 overexpression and in 293 T cells. ****p < 0.0001.

In order to investigate the impact of p53 on PC progression, we stably overexpressed p53 in PANC‐1 and BxPC‐3 cells through lentiviral infection. The successful overexpression was confirmed by qRT‐PCR and western blotting analysis (Figure 4A–F). In addition, we stably transduced PANC‐1 and BxPC‐3 cells with lentiviral shRNA constructs targeting p53 to knock down its expression effectively (Figure 4G–J). In order to determine whether A2M‐AS1 is a crucial downstream gene regulated by p53 in PC cell migration and invasion, we conducted lentivirus‐mediated A2M‐AS1‐shRNA knockdown to reduce its expression in p53‐overexpressing cells, as well as induced endogenous expression of A2M‐AS1 in stable p53‐knockdown cells (Figure 4K,L). Subsequently, we examined the levels of A2M‐AS1 expression in both p53‐overexpressing and knockdown cells. The results indicated that p53 inhibited the expression of A2M‐AS1 significantly (Figure 4M,N). Next, our aim was to elucidate the mechanism by which p53 regulates A2M‐A21 gene expression. Firstly, we analyzed the upstream promoter sequence of the A2M‐A21 gene using bioinformatics. Our findings revealed a binding site for p53 within this region (Figure 4O,P). Furthermore, a dual‐luciferase reporter assay was performed to confirm transcriptional regulation of the A2M‐A21 gene by p53. The results demonstrated that overexpression of p53 increased promoter activity for this gene (Figure 4Q).

### 3.5 p53 Depression Amplified A2M‐AS1 Inhibition of PC Progression

**FIGURE 5:**
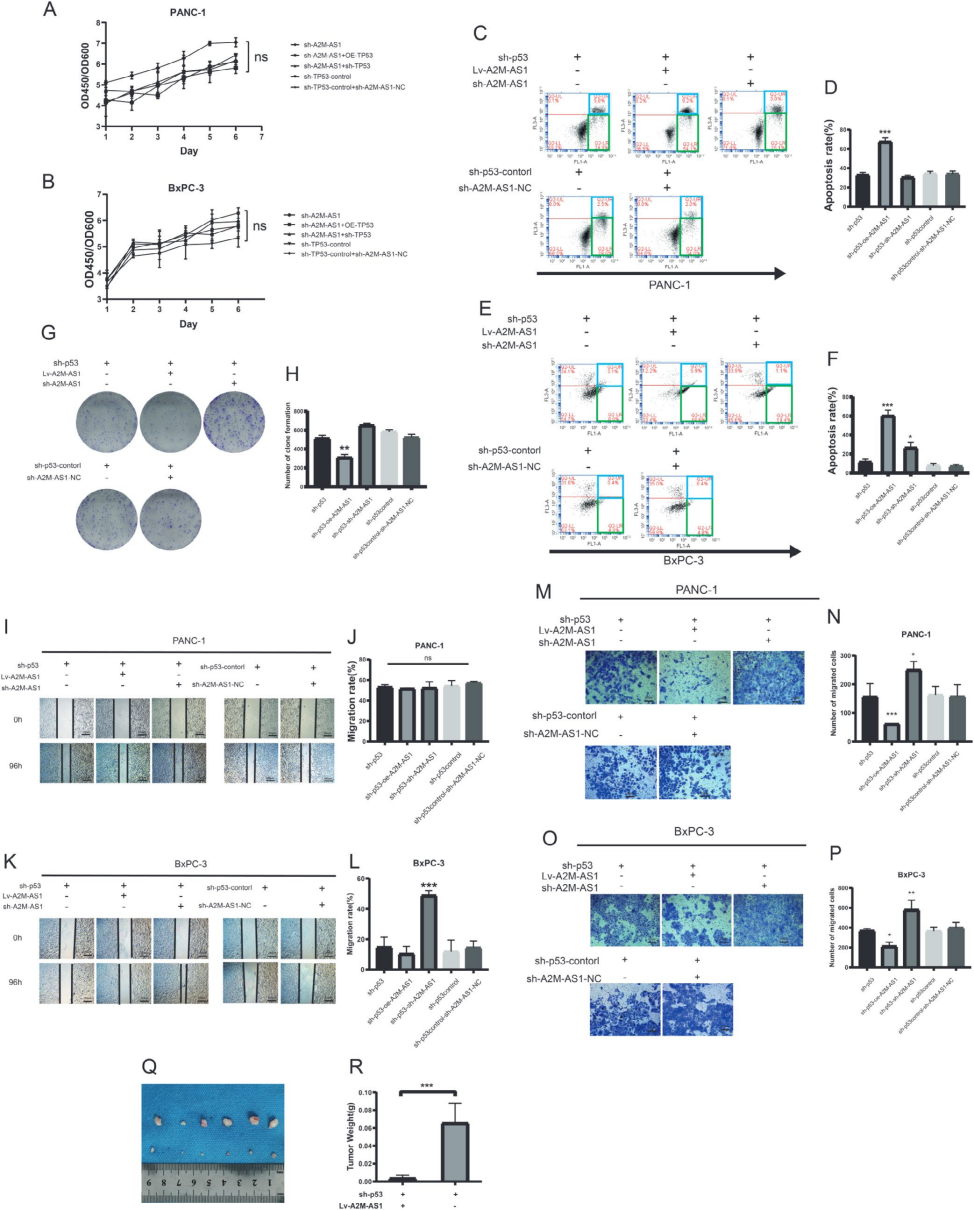
p53 depression amplified A2M‐AS1 inhibition of pancreatic cancer progression. (A, B) The effect of ectopic expression/knockdown p53 on A2M‐AS1 overexpression/knockdown proliferation. ns > 0.05. (C, D) Apoptosis assay following ectopic expression/knockdown p53 based on A2M‐AS1 overexpression/knockdown in PANC‐1. ***p < 0.001. (E, F) Apoptosis assay following ectopic expression/knockdown p53 based on A2M‐AS1 overexpression/knockdown in BxPC‐3. ***p < 0.001. (G, H) Clone formation assay following ectopic expression/knockdown p53 based on A2M‐AS1 overexpression/knockdown in PANC‐1. **p < 0.01. (I, J) Scratch assay following ectopic expression/knockdown p53 based on A2M‐AS1 overexpression/knockdown in PANC‐1. Scale bars, 200 μm. ns > 0.05. (K, L) Scratch assay following ectopic expression/knockdown p53 based on A2M‐AS1 overexpression/knockdown in BxPC‐3. Scale bars, 200 μm. ***p < 0.001. (M, N) The effect of ectopic expression/knockdown p53 based on A2M‐AS1 overexpression/knockdown on PANC‐1 invasion. Scale bars, 200 μm. *p < 0.05, ***p < 0.001. (O, P) The effect of ectopic expression/knockdown p53 based on A2M‐AS1 overexpression/knockdown on BxPC‐3 invasion. Scale bars, 200 μm. *p < 0.05, **p < 0.01. (Q, R) PANC‐1 cells infected with either A2M‐AS1 overexpression or control virus based on knockdown p53 were subcutaneously implanted into randomized nude mice to construct a xenograft model (five mice per group). The tumor developments were quantified based on the liver weight (right). Data are shown as the mean ± SD. Two‐tailed *t*‐test. ***p < 0.001.

Following elucidating the regulatory relationship between p53 and A2M‐AS1 in PC cells, we aimed to investigate the pivotal role of p53‐A2M‐AS1 in PC progression. Proliferation assays demonstrated that ectopic expression or knockdown of both p53 and A2M‐AS1 did not significantly affect the proliferation of PC cells (Figure 5A,B). However, simultaneous knockdown of p53 and overexpression of A2M‐AS1 induced apoptosis in PC cells, as evidenced by flow cytometric analysis results (Figure 5C–F). Colony formation assays revealed that p53 ectopic expression and knockdown of A2M‐AS1 inhibited clonogenicity in PC cells (Figure 5G,H). Wound healing assays indicated that the downregulation of p53 and A2M‐AS1 promoted migration in BxPC‐3 cells but did not impact PANC‐1 cells (Figure 5I–L). Transwell assays demonstrated that simultaneous knockdown of p53 with overexpression of A2M‐AS1 significantly attenuated invasion capability in PC cells, while knockdown alone enhanced invasion potential (Figure 5M–P). Furthermore, to further elucidate the role played by p53‐A2M AS1 in PC metastasis in vivo, we established a subcutaneous tumor model using nude mice. Lentivirus‐mediated overexpression of p53 and A2M‐AS1 knockdown were performed on PANC‐1 cells, which exhibited an augmented ability to promote tumor growth (Figure 5Q,R). Collectively, these data indicated that the knockdown of p53 can amplify A2M‐AS1 inhibition of PC.

### 3.6 p53‐A2M‐AS1 Modulates PC Progression by the MAPK Pathway

**FIGURE 6:**
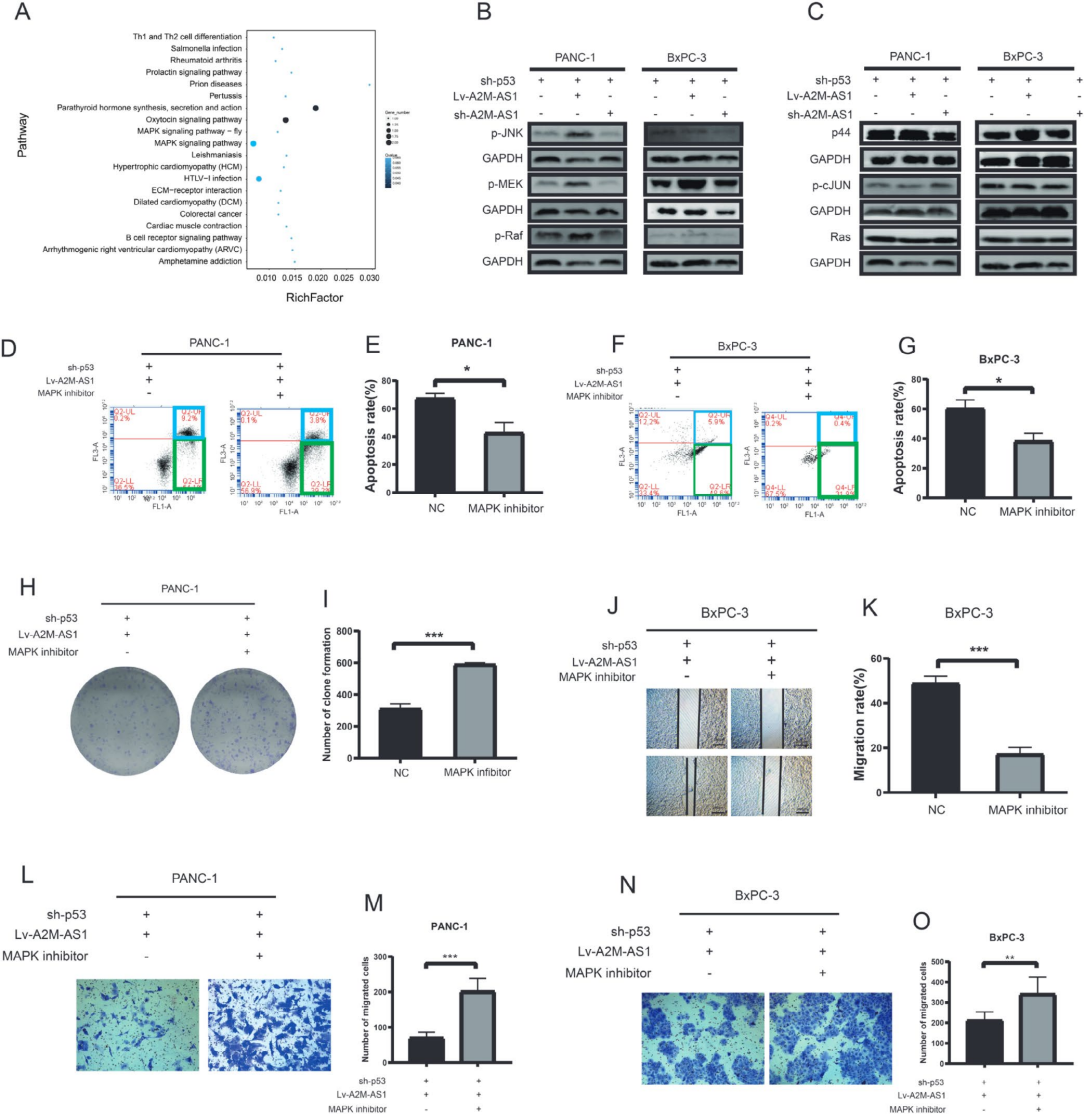
p53‐A2M‐AS1 modulates pancreatic cancer progression by MAPK pathway. (A) Scatterplot of the upregulated gene expression clusters in control (Ctrl) and A2M‐AS1‐overexpressing (OE) PANC‐1 cells after KEGG enrichment analysis. (B, C) A2M‐AS1 overexpression and silencing based on knockdown p53 activated MAPK signaling pathways. (D, E) The effect of MAPK inhibitor on A2M‐AS1 overexpression and p53 silencing in PANC‐1. *p < 0.05. (F, G) The effect of MAPK inhibitor on A2M‐AS1 overexpression and p53 silencing in BxPC‐3. *p < 0.05. (H, I) Clone formation assay following MAPK inhibitor co‐culture with A2M‐AS1 overexpression and p53 silencing in PANC‐1. ***p < 0.001. (J, K) Scratch assay following MAPK inhibitor co‐culture with A2M‐AS1 overexpression and p53 silencing in BxPC‐3. Scale bars, 200 μm.***p < 0.001. (L, M) The effect of MAPK inhibitor co‐culture with A2M‐AS1 overexpression and p53 silencing in PANC‐1. Scale bars, 200 μm. ***p < 0.001. (N, O) The effect of MAPK inhibitor co‐culture with A2M‐AS1 overexpression and p53 silencing in BxPC‐3. Scale bars, 200 μm. **p < 0.01.

In order to explore the mechanism underlying A2M‐AS1 inhibited PC progression, we overexpressed A2M‐AS1 in PANC‐1 cells and performed gene sequencing. Compared with the reference genome and reconstructed transcripts, 26,159 novel transcripts were identified. Among them, 24,152 represented new variable splicing subtypes of known protein‐coding genes, 240 were transcripts of newly discovered protein‐coding genes, and 1767 were long‐chain lncRNAs. KEGG enrichment analysis on the downstream differentially expressed genes indicated significant alterations in the MAPK pathway (Figure 6A). Consequently, we conducted further experiments to validate the involvement of the MAPK pathway in PC cells. Western blotting experiments demonstrated that silencing p53 and overexpressing A2M‐AS1 in PANC‐1 and BxPC‐3 PC cell lines led to increased phosphorylation levels of the JNK, MEK, and Raf proteins, which are key proteins of the MAPK pathway. Conversely, knockdown of A2M‐AS1 and silencing of p53 resulted in decreased phosphorylation levels of the JNK, MEK, and Raf proteins (Figure 6B). The expression levels of the p44 protein, Ras total protein, and c‐JUN phosphorylated protein remained unchanged upon overexpression or knockdown of A2M‐AS1 following p53 silencing in PC cell lines (Figure 6C). These findings suggest that p53 silencing and overexpression of A2M‐AS1 can activate the MAPK pathway by promoting phosphorylation within its associated proteins while both knockdown of p53 and A2M‐AS1 could inhibit MAPK pathway activation.

**FIGURE 7:**
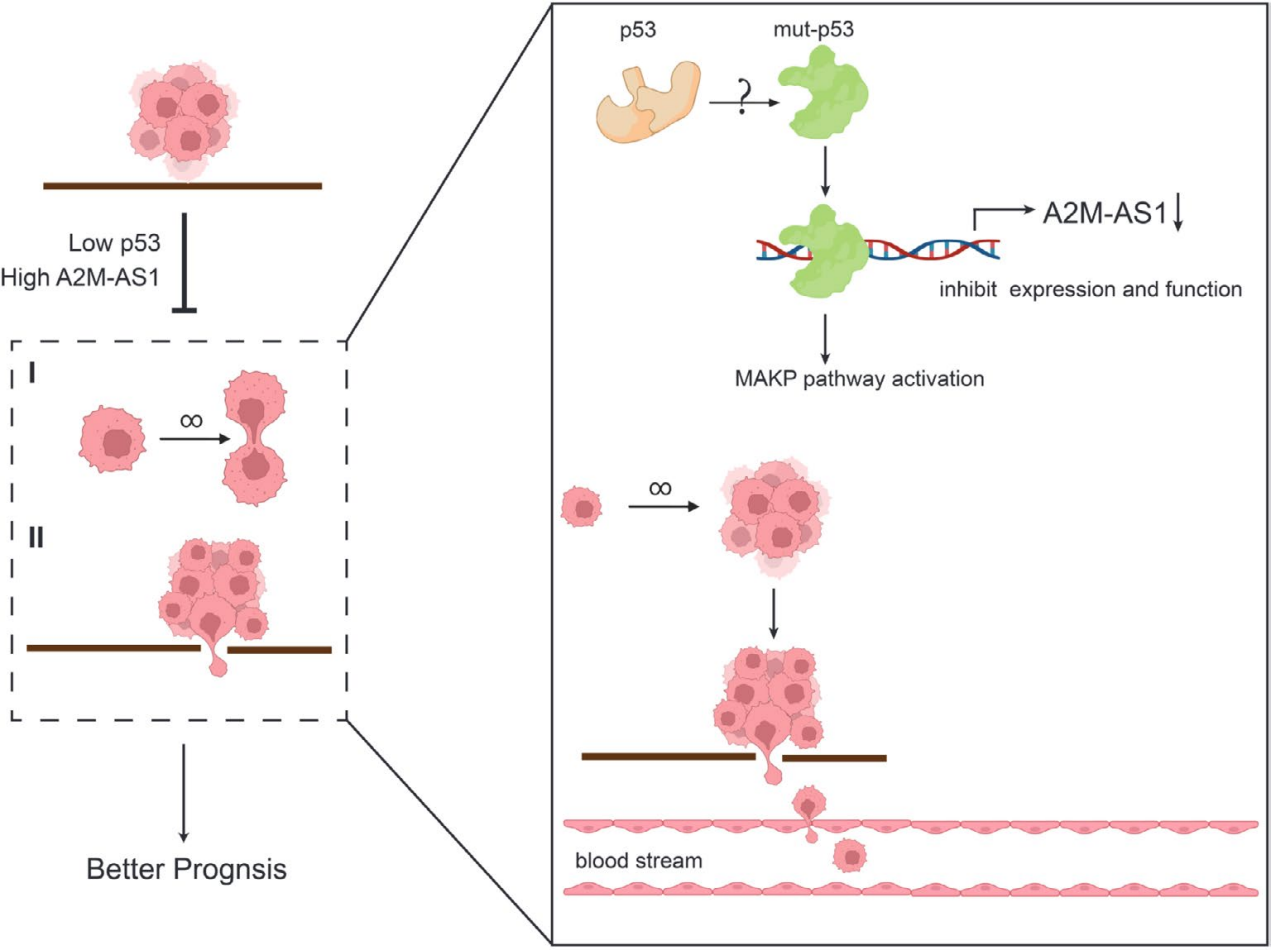
Proposed model for the role of p53‐A2M‐AS1 on PC progression. In pancreatic cancer, p53 functions as an accelerated tumor factor by suppressing A2M‐AS1 expression and function, which binds to the A2M‐AS1 promoter gene sequence, then activates the MAPK pathway and ultimately influences the patient outcome. p53 low expression and A2M‐AS1 high expression A2M‐AS1 attenuates tumor growth and metastasis. Nevertheless, different from p53 in 293 T cells, p53 mutation may caused inhibition of A2M‐AS1 in pancreatic cancer, which remains be illuminated.

In order to investigate the role of the MAPK pathway as a critical downstream signaling pathway of p53‐A2M‐AS1 in regulating the progression of PC cells, we employed a MAPK inhibitor to effectively block this pathway. The proliferation assays demonstrated that inhibiting the MAPK pathway was sufficient to abolish p53‐A2M‐AS1‐induced apoptosis in PC cells (Figure 6D–G). Furthermore, colony formation assays revealed that treatment with the MAPK inhibitor alleviated the inhibitory effect on colony‐forming ability caused by p53‐A2M‐AS1 (Figure 6H,I). Transwell and wound healing assays further confirmed that the inhibition of the MAPK pathway significantly reversed the suppressive effects mediated by p53‐A2M‐AS1 on the invasion and migration abilities of PC cells (Figure 6J–O). Collectively, these findings underscored the functional indispensability of the MAPK pathway in mediating p53‐A2M‐AS1‐driven development of PC cells (Figure 7).

## 4 Discussion

Despite significant advancements in medical and healthcare, the treatment outcomes for patients with pancreatic ductal adenocarcinoma (PDAC) remain poor [9]. PDAC has now surpassed breast cancer as the third leading cause of cancer‐related mortality due to its increasing incidence, late clinical diagnosis, and limited therapeutic options [10]. While surgery remains the primary treatment modality for PC, it is predominantly suitable for early‐stage patients. Unfortunately, a majority of PDAC patients are diagnosed at an advanced stage when surgical intervention is no longer feasible due to tumor metastasis or other complications [11]. Consequently, conventional cytotoxic chemotherapy regimens are often employed but exhibit restricted efficacy and high drug toxicity, coupled with resistance toward targeted therapies, resulting in a dismal 10‐year survival rate of only 1% [12]. With the continuous rise in PDAC incidence over time, it is anticipated that by 2030, it will become the second leading cause of cancer‐related mortality [13], underscoring the urgent need for novel treatment strategies.

The p53 protein and its association with cancer have been extensively investigated since its initial discovery in 1979 [14]. As a transcription factor, p53 regulates the expression of target genes and plays a crucial role in cellular processes such as cell cycle arrest, apoptosis, DNA repair, and other biological activities [15]. However, tumor cells often harbor various *TP53* gene mutations that primarily result in missense mutations associated with cancer [16]. These mutations lead to substituting a single amino acid within the protein structure [17]. Some cases with intense positive p53 staining within tumor tissues are typically attributed to *TP*53 mutation‐induced overexpression of a non‐functional p53 protein [18]. PC is often related to *TP53* mutations, and about 70% of PCs have mutations inactivating *TP53* [19], but such mutations are late events in PC pathogenesis [20, 21]. Still, the present study only examined p53 overexpression or silencing and did not evaluate the impact of *TP53* mutations on A2M‐AS1. It will have to be investigated in the future.

Our findings revealed significantly higher levels of p53 expression in PC tissues compared with adjacent noncancerous tissues, whereas A2M‐AS1 expression was significantly lower in PC tissues. p53 and A2M‐AS1 were associated with patient prognosis and could be reliable prognostic markers for PC. Furthermore, patients with low p53 and high A2M‐AS1 expression had a significantly longer survival time than the other groups. In addition, the TCGA results were consistent with those of the patients included in this study. The results suggest that high expression of p53 is associated with a poor PC prognosis, consistent with the literature [22, 23]. Hence, it can be speculated that the overexpression of p53 in PC is likely attributed to *TP53* mutation, leading to the loss of its original protein function [22]. Furthermore, by integrating TCGA database analysis, we observed an association between p53 and A2M‐AS1 expression and its impact on the unfavorable prognosis of PC. Our findings suggest that p53 is negatively associated with A2M‐AS1, and p53 alterations intricately changed the biological role of A2M‐AS1 in PC.

In this study, there were no significant differences in the proliferation rate of PANC‐1 and BxPC‐3 cells among all groups. However, silencing p53 and knockdown A2M‐AS1 significantly inhibited the clonogenic ability of PANC‐1 cells in the colony formation assay, indicating that silencing p53 and knocking down A2M‐A21 had a profound impact on the transient stability of PC cells. After transfection, PC cells exhibited increased sensitivity to processes such as enzymatic digestion and implantation within a short period, resulting in slower recovery rates during this time frame. Conversely, in the colony formation assay, where cells are allowed to remain for an extended duration, they had sufficient time to recover their biological state, leading to a more pronounced proliferative trend. Furthermore, during daily cell culture procedures, it was noted that PANC‐1 and BxPC‐3 cells subjected to p53 silencing and A2M‐A21 knockdown exhibited enhanced proliferation compared with the other groups. However, these cells displayed greater morphological instability. Due to alterations in biological function following double transfection attempts with BxPC‐3 cells, many unsuccessful cloning attempts were made without a clear understanding of the exact underlying reasons.

We examined the expression level of p53 on A2M‐AS1 and observed that the overexpression of p53 suppressed the expression of A2M‐AS1 in PC cells, whereas silencing of p53 increased the expression level of A2M‐AS1. However, results from dual luciferase reporter gene assays demonstrated that p53 enhanced the promoter activity of the A2M‐AS1 gene, suggesting that overexpressing p53 in 293 T cells could potentially upregulate the expression of A2M‐AS1. The contradictory findings can likely be attributed to several factors. As mentioned above, the transfected cells used in the dual luciferase reporter gene experiment were 293 T cells with normal p53 protein expression. However, in PC cells, various carcinogenic factors may lead to mutations in the p53 gene and post‐translational modifications that result in the loss or even acquisition of abnormal function by mutant p53 proteins, promoting cancer development. In addition, conducting a dual luciferase assay after transfecting both p53 and A2M‐AS1 silencing plasmids would provide further insights into their regulatory effects.

We identified differentially expressed genes through enrichment analysis using the KEGG pathway database. By predicting downstream pathways regulated by p53‐A2M‐AS1 in PC cells, we specifically observed significant alterations in the MAPK pathway. Interestingly, A2M‐AS1 overexpression and p53 silencing resulted in the activation of multiple protein phosphorylation events within the MAPK pathway, suggesting a potential regulatory role for P53‐A2M‐AS1 in PC cells. Moreover, rescue experiments utilizing MAPK blockers demonstrated their ability to reverse the biological changes induced by the overexpression of A2M‐AS1 after silencing p53.

The in vivo data contradicted the in vitro data. Such a phenomenon is not uncommon since in vitro assays are performed with a pure population of cells in a tightly controlled environment dictated by the culture medium. On the other hand, in in vivo experiments, the cancer cells are influenced by the tumor microenvironment and by all the cytokines, growth factors, hormones, etc., found in a living organism [24, 25, 26]. Additional studies are necessary to investigate the role of A2M‐AS1 in PC.

In conclusion, high A2M‐AS1 expression is associated with a better PC prognosis, particularly with low p53 expression. A2M‐AS1 overexpression subdues PC cell development. Our study unveils a new mechanism by which p53 decreases A2M‐AS1 expression.

## Author Contributions

**Yu Cen:** data curation (equal), formal analysis (equal), investigation (equal), methodology (equal), software (equal), writing – original draft (equal). **Yihui Luo:** data curation (equal), investigation (equal), methodology (equal). **Yi Nan:** data curation (equal), investigation (equal), methodology (equal). **Wenxi Kuang:** data curation (equal), investigation (equal), methodology (equal). **Xianglian Zhang:** data curation (equal), investigation (equal), methodology (equal). **Jiexia Lu:** data curation (equal), investigation (equal), methodology (equal). **Chunxiao Xie:** data curation (equal), investigation (equal), methodology (equal). **Mingzhi Xie:** data curation (equal), investigation (equal), methodology (equal). **Enran Chen:** funding acquisition (equal), investigation (equal), methodology (equal). **Haixing Jiang:** conceptualization (equal), formal analysis (equal), project administration (equal), supervision (equal), visualization (equal), writing – review and editing (equal). **Shanyu Qin:** conceptualization (lead), formal analysis (equal), funding acquisition (lead), project administration (equal), resources (lead), supervision (equal), validation (equal), visualization (equal), writing – review and editing (equal).

## Ethics Statement

Ethical approval was given by the Ethics Committee for Animal Use of Guangxi Medical University (#2024‐E305‐01).

## Consent

The authors have nothing to report.

## Conflicts of Interest

The authors declare no conflicts of interest.

## Supporting information


Data S1.

**Table S1.** Clinical baseline data of pancreatic cancer patients and the correlation between the expression levels of p53 and A2M‐AS1 and clinical characteristics of pancreatic cancer patients.

## Data Availability

The datasets used and/or analyzed during the current study are available from the corresponding author upon reasonable request.
