# Supplementary material for: Knockdown of p53 Enhances LncRNA A2M‐AS1 Inhibition of Pancreatic Cancer Progression via Regulating MAPK Pathway
Source: Cancer Med. 2025 Jul 13;14(13):e70956. doi: 10.1002/cam4.70956 (PMC12256274; doi:10.1002/cam4.70956)

FISH

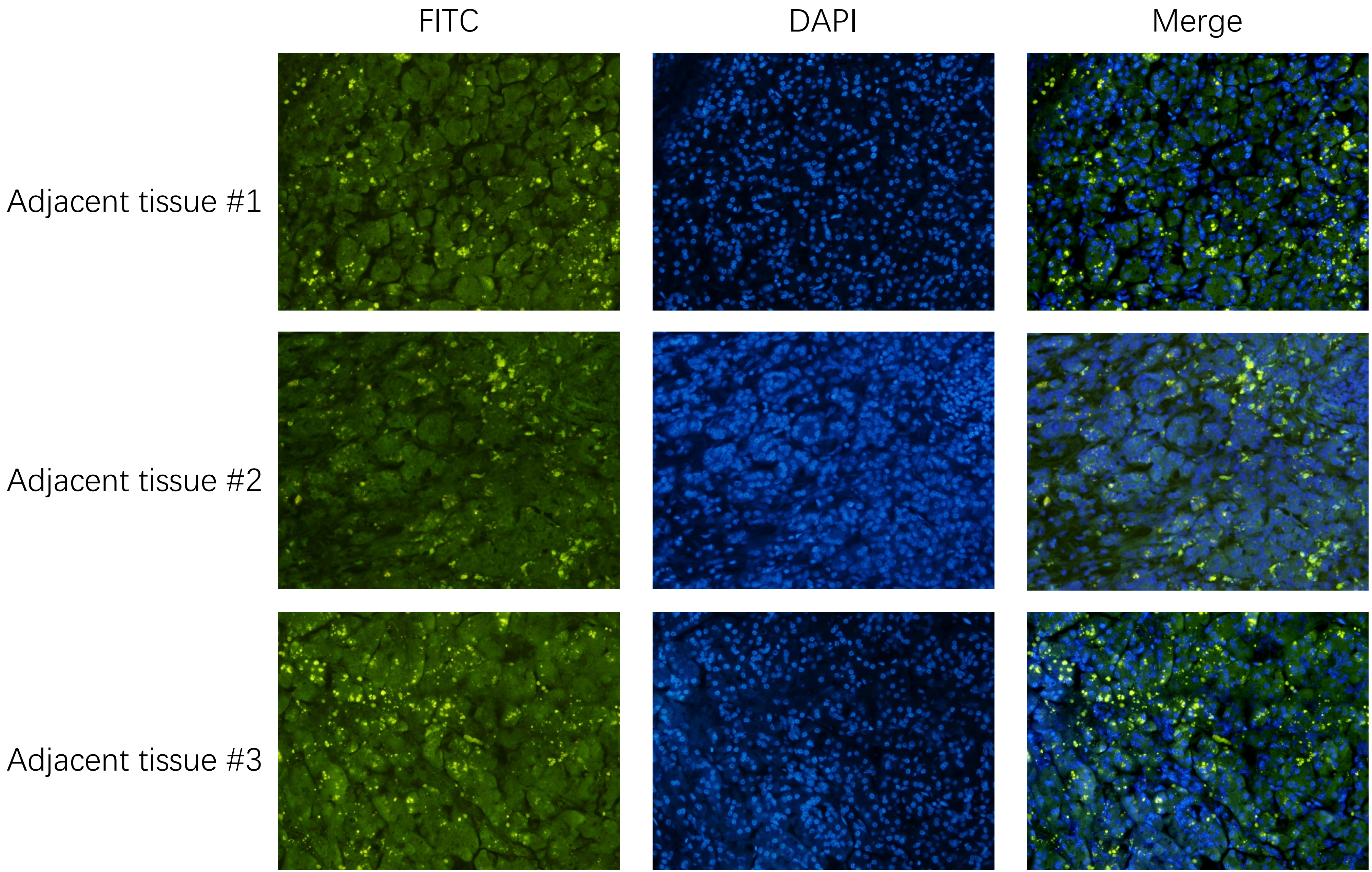
FITC
DAPI
Merge
Adjacent tissue #1
Adjacent tissue #2
Adjacent tissue #3

Transfection efficiency

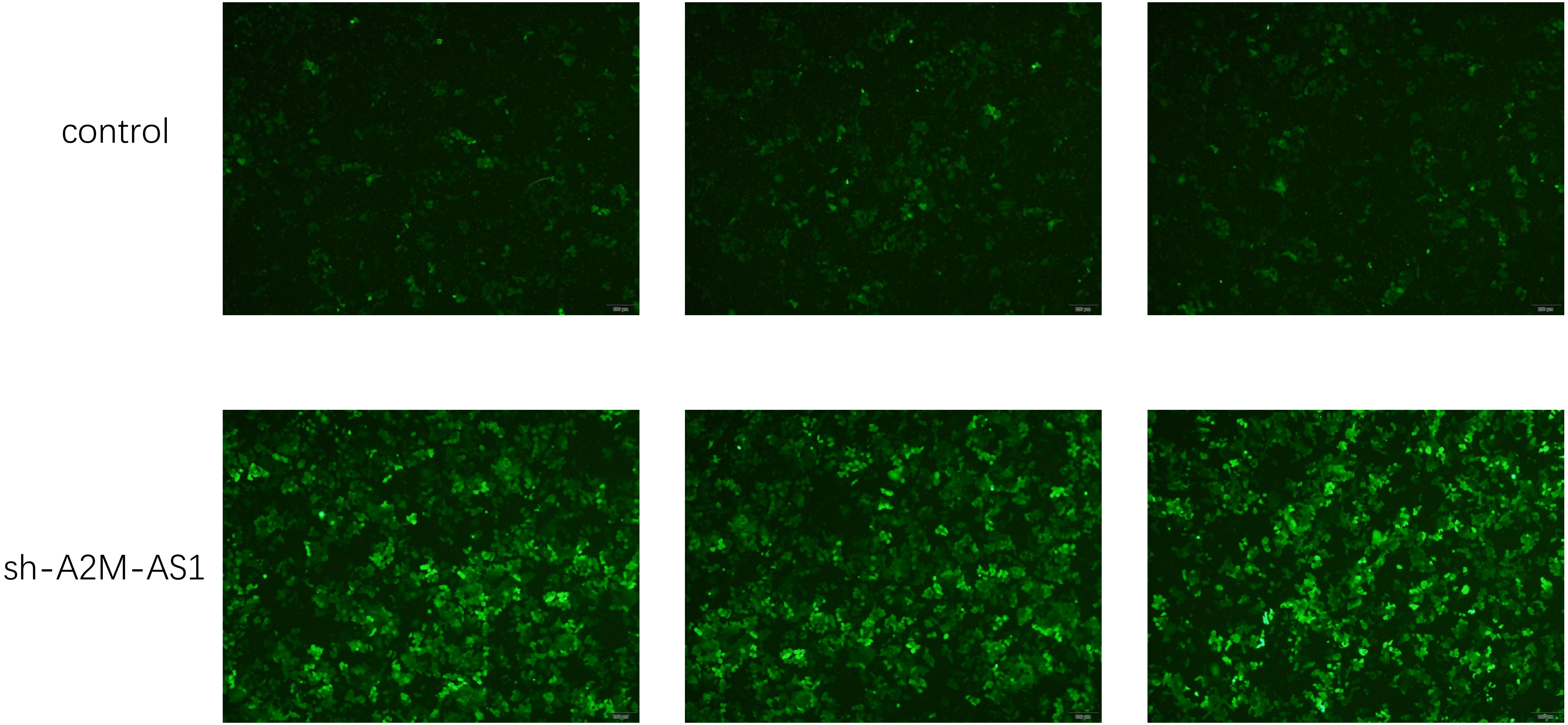

Migration

control

0 h

72 h

sh-A2M-AS1

0 h

72 h

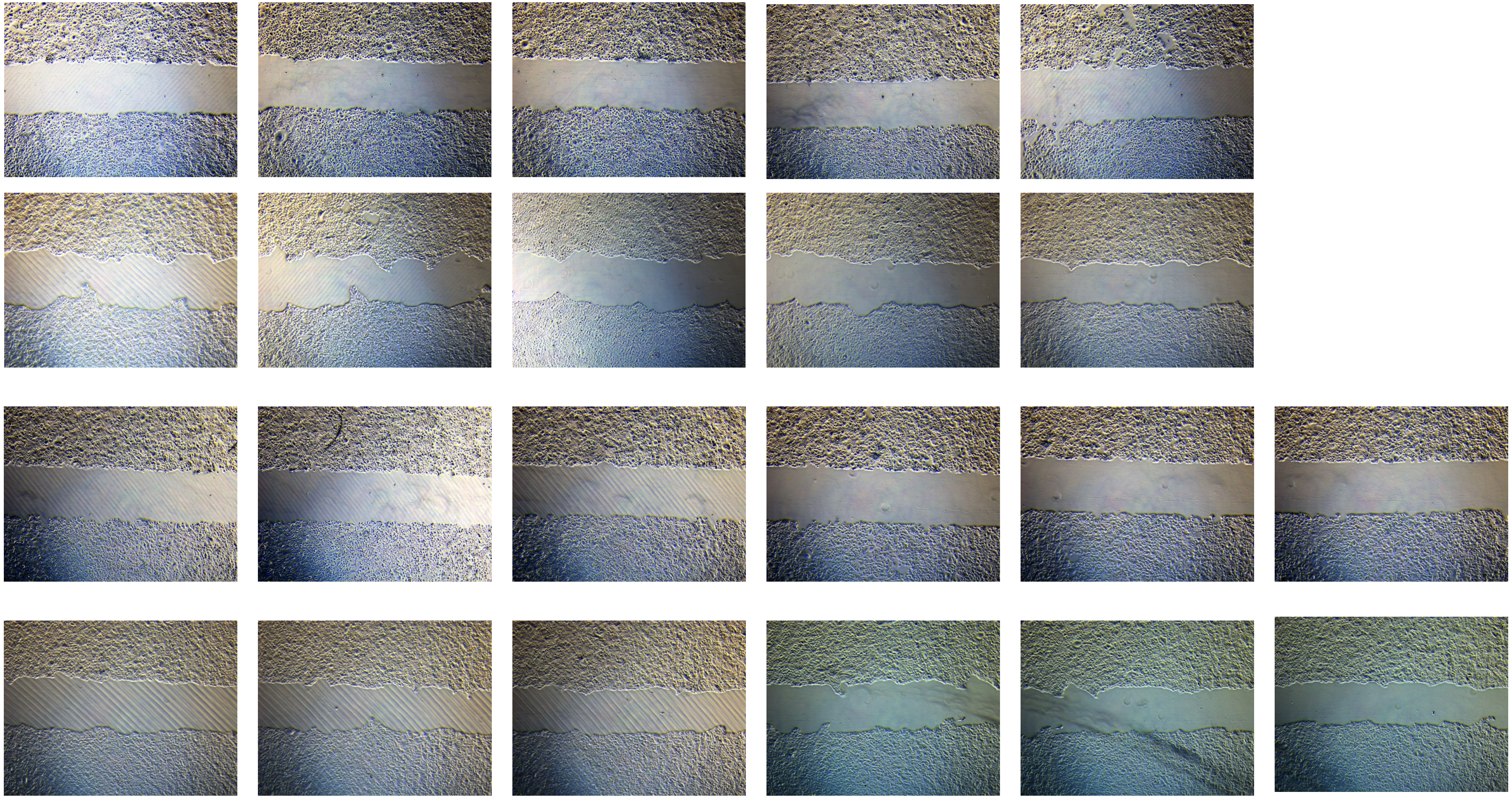

Invasion

control

sh-A2M-AS1

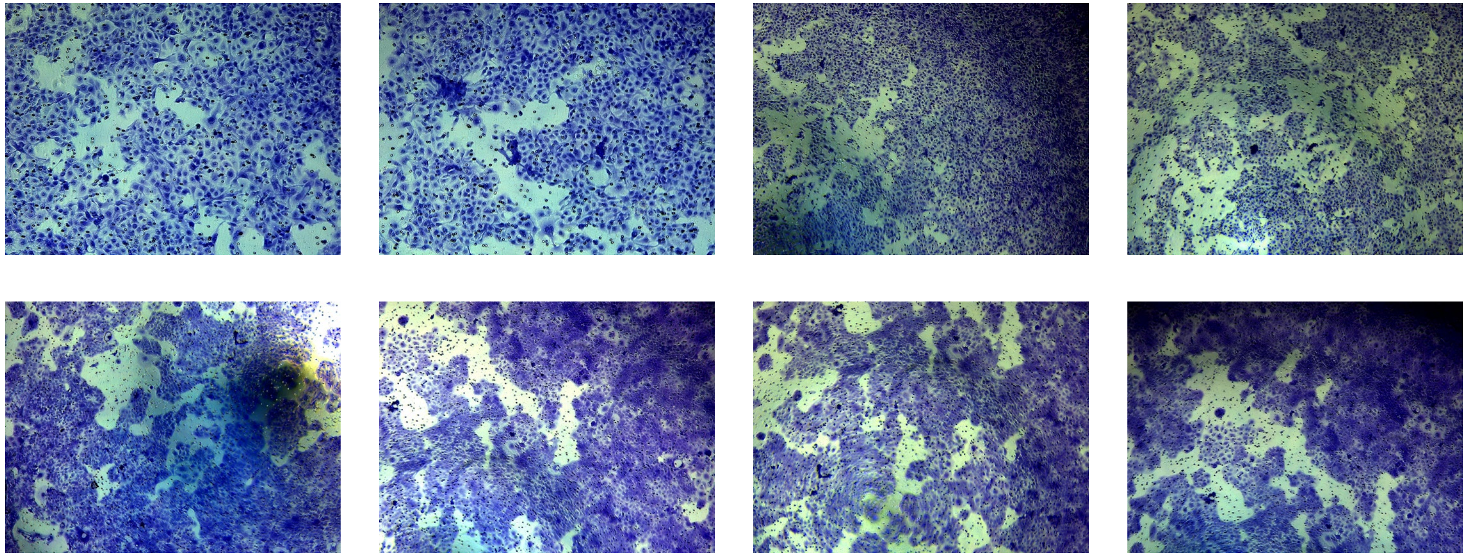

WB results with control

Target protein: P-JNK

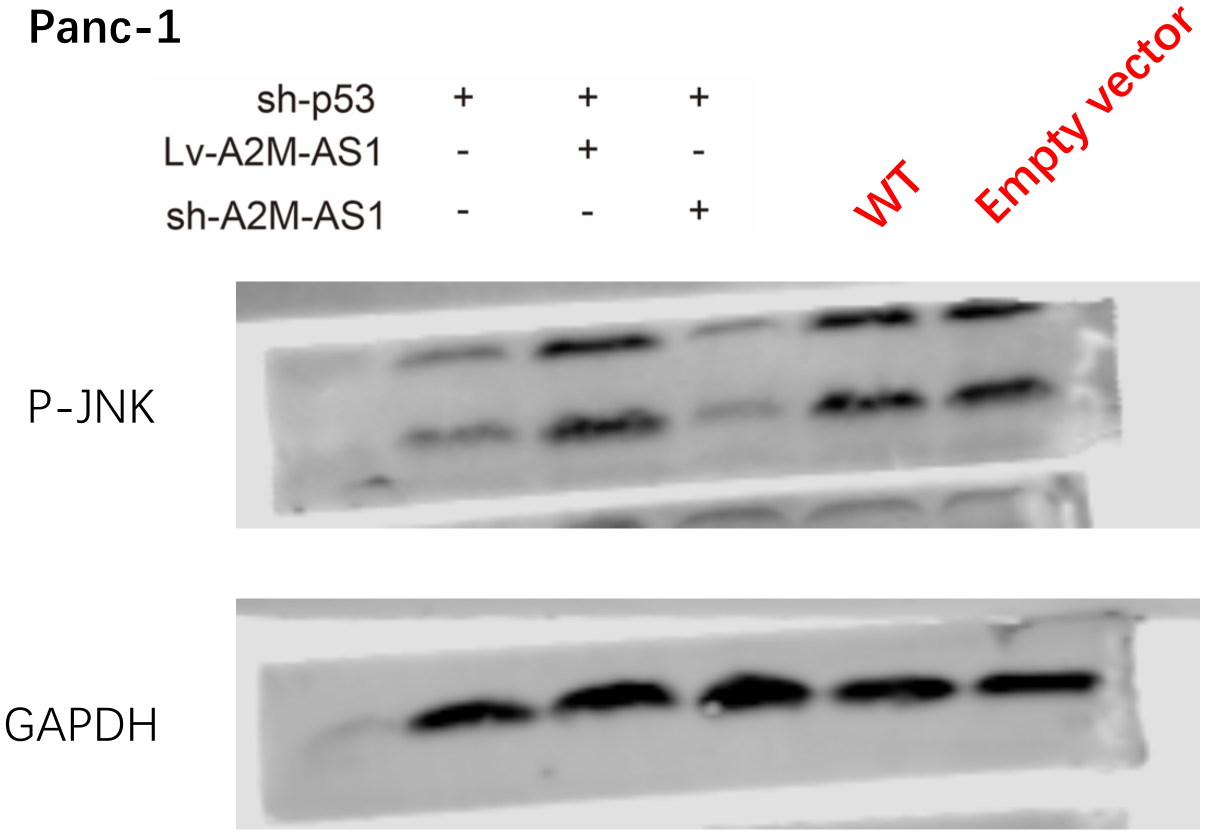

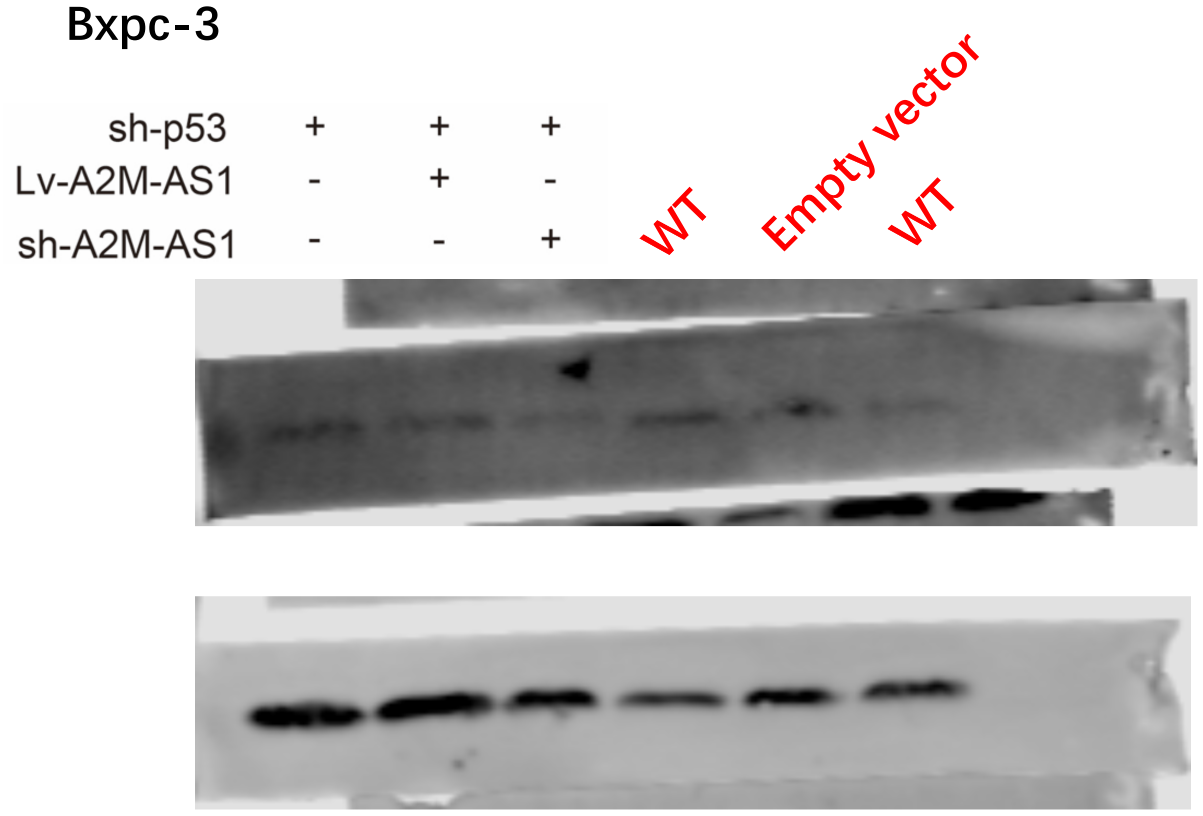

WB results with control

Target protein: P-MEK

Panc-1

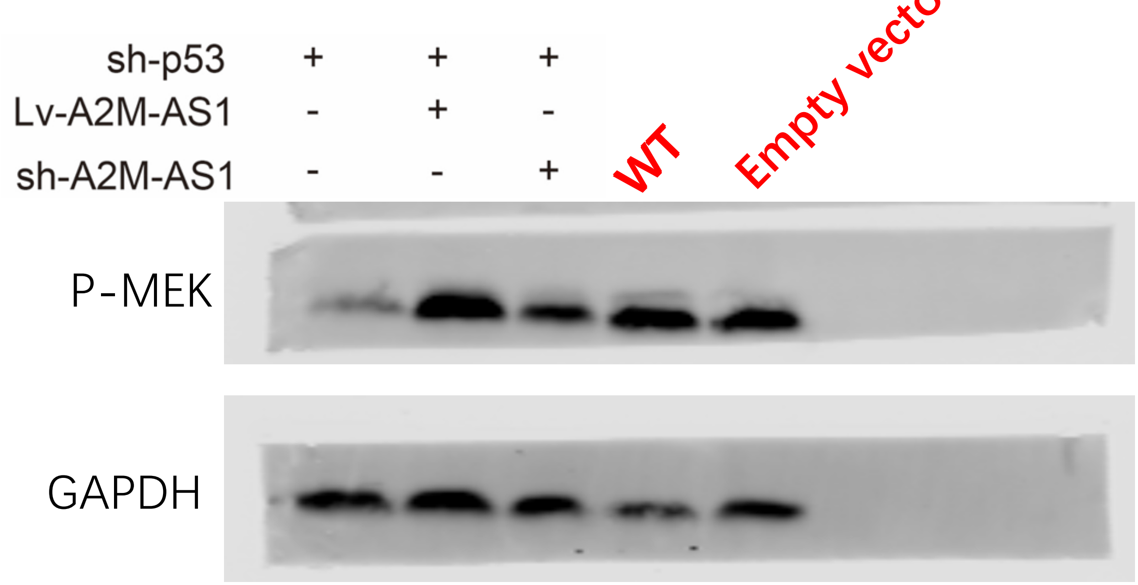

Bxpc-3

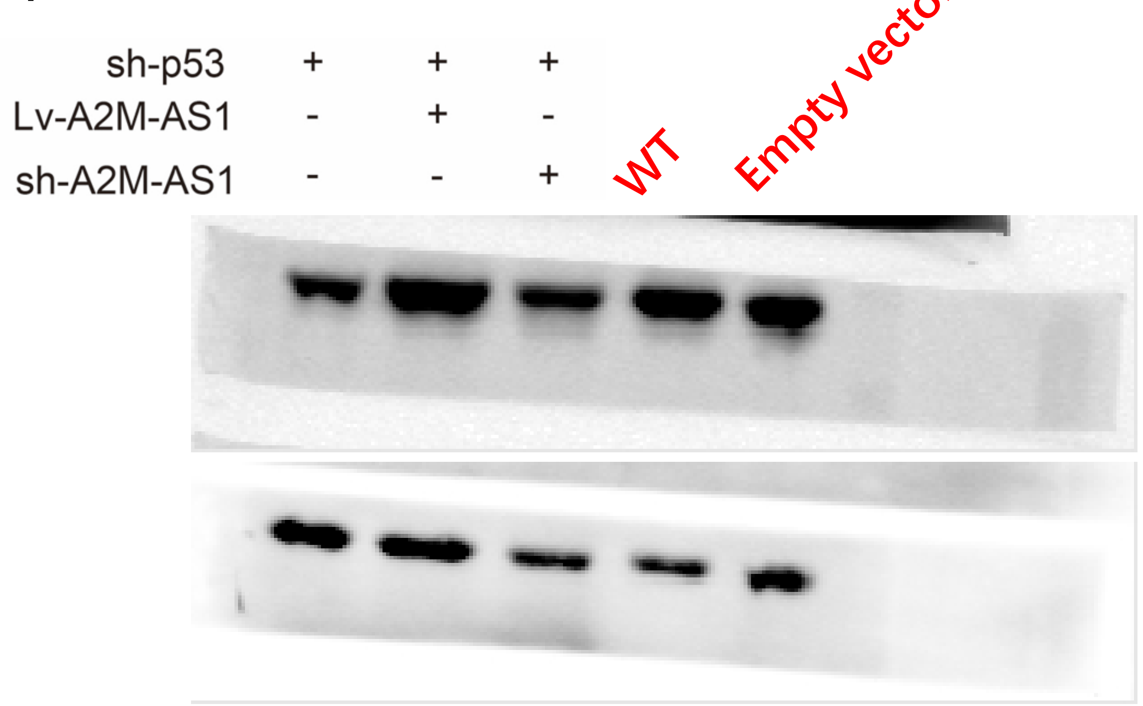

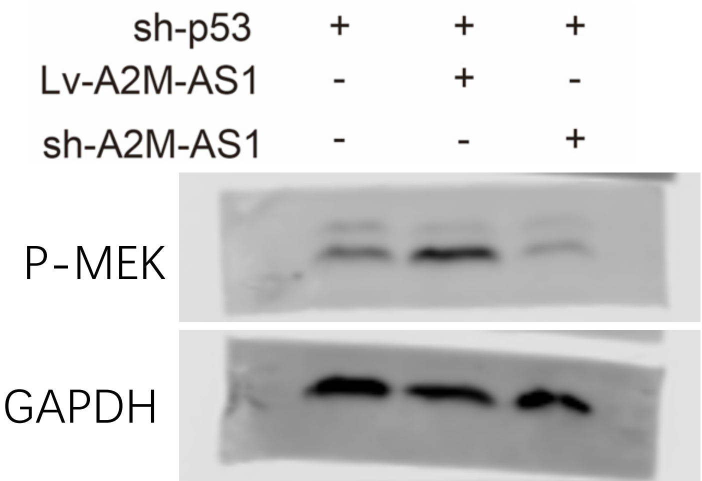

WB results with control

Target protein: P-Raf

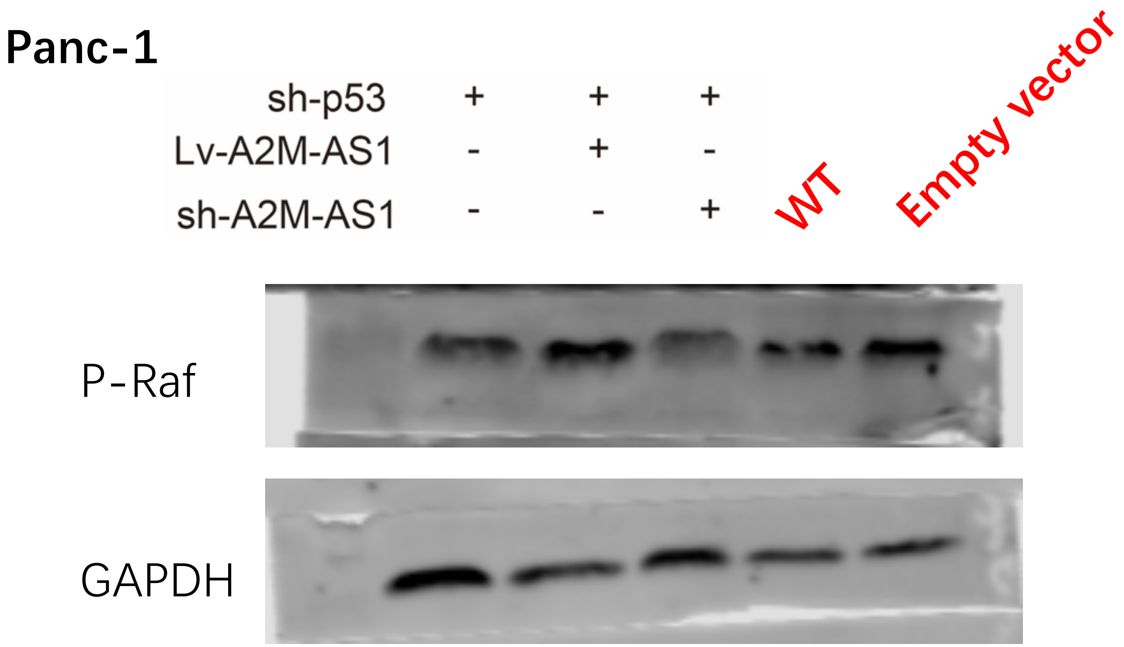

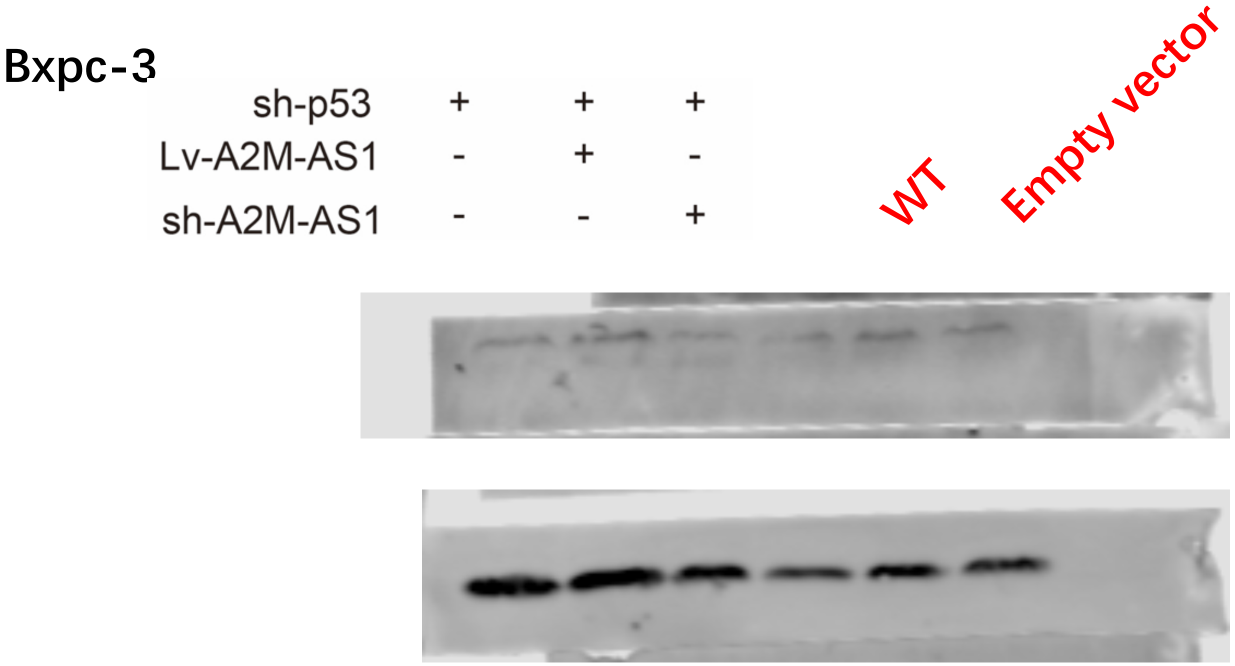

WB results with control

Target protein: P-p44

Panc-1

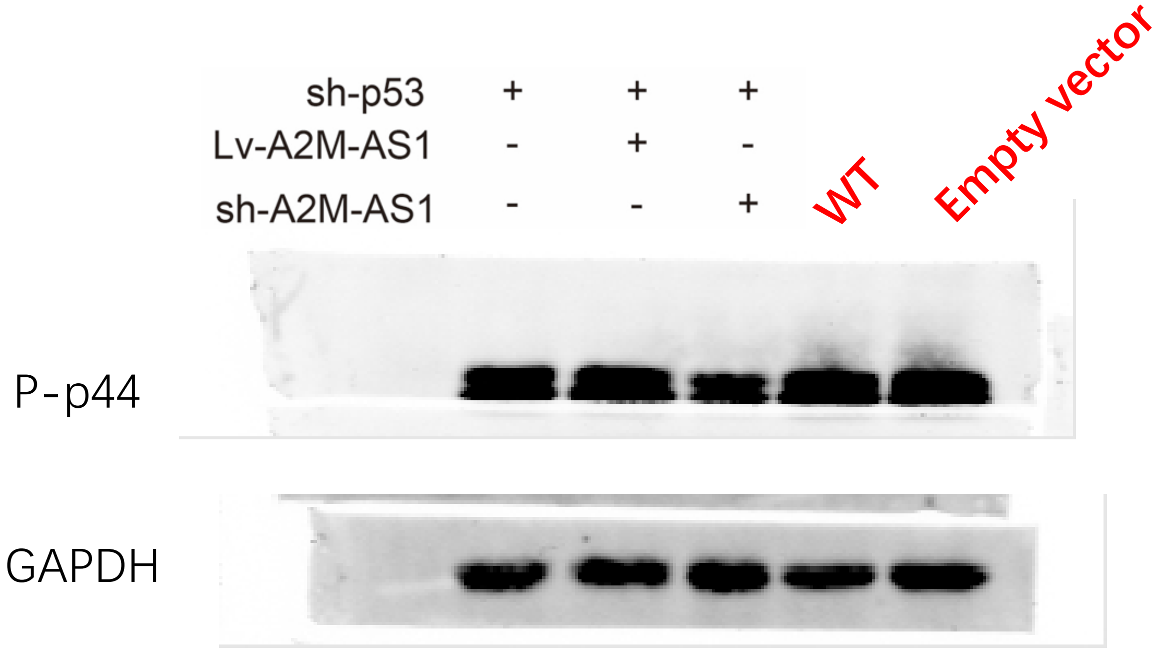

Bxpc-3

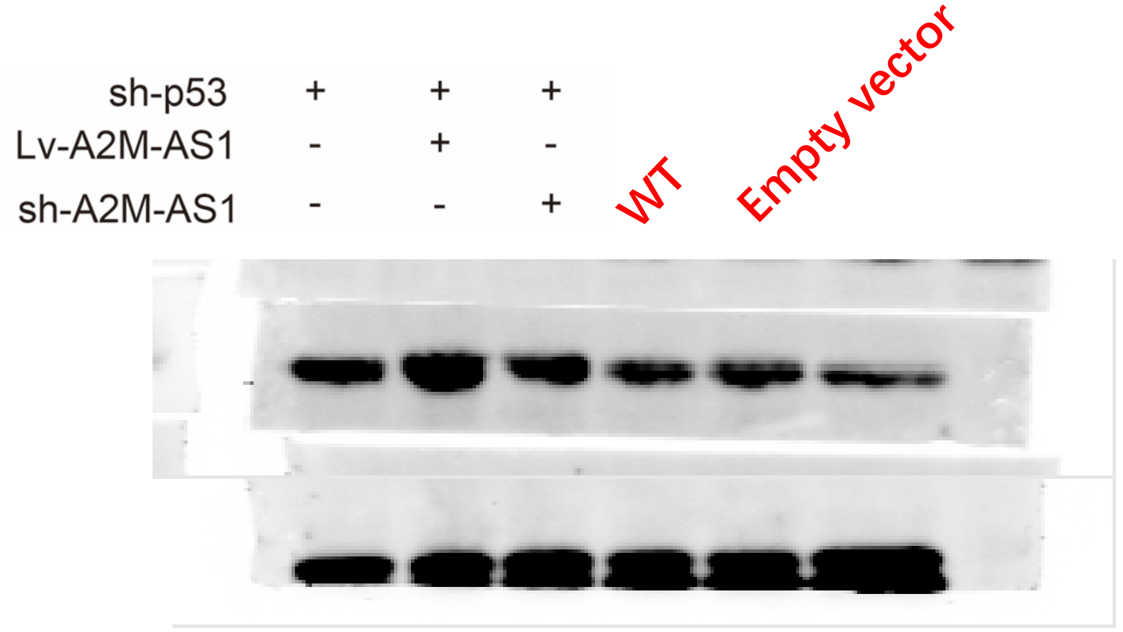

WB results with control

Target protein: P-JUN

Panc-1

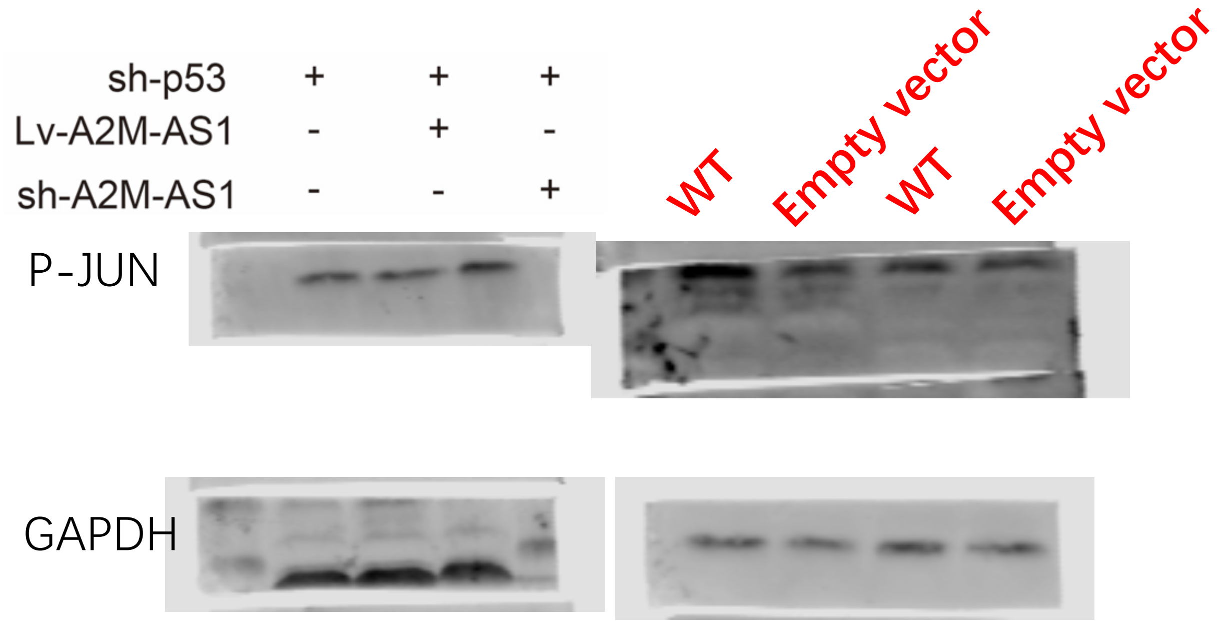

Bxpc-3

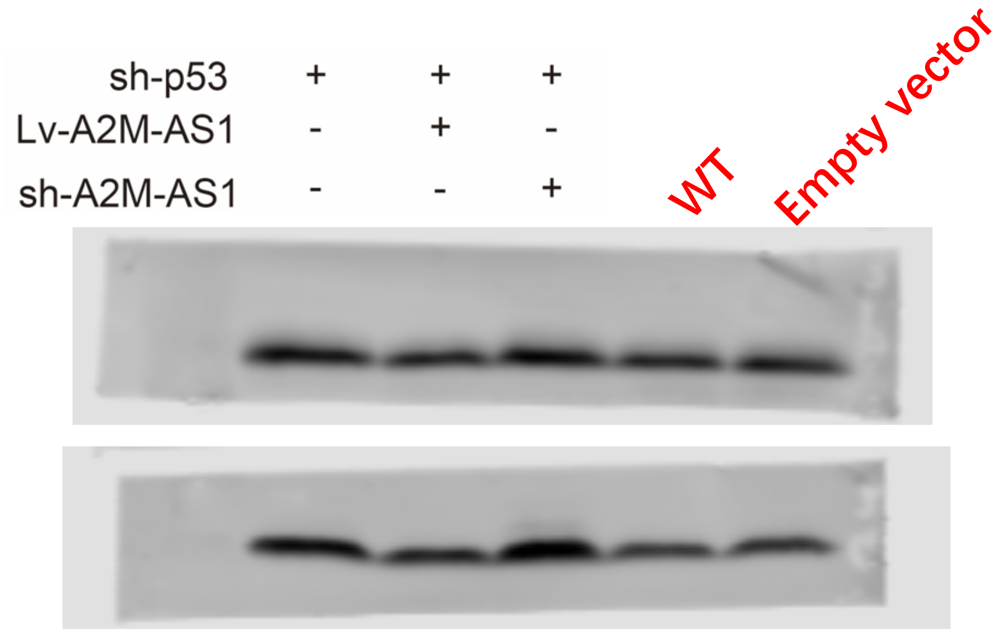

WB results with control

Target protein: Ras

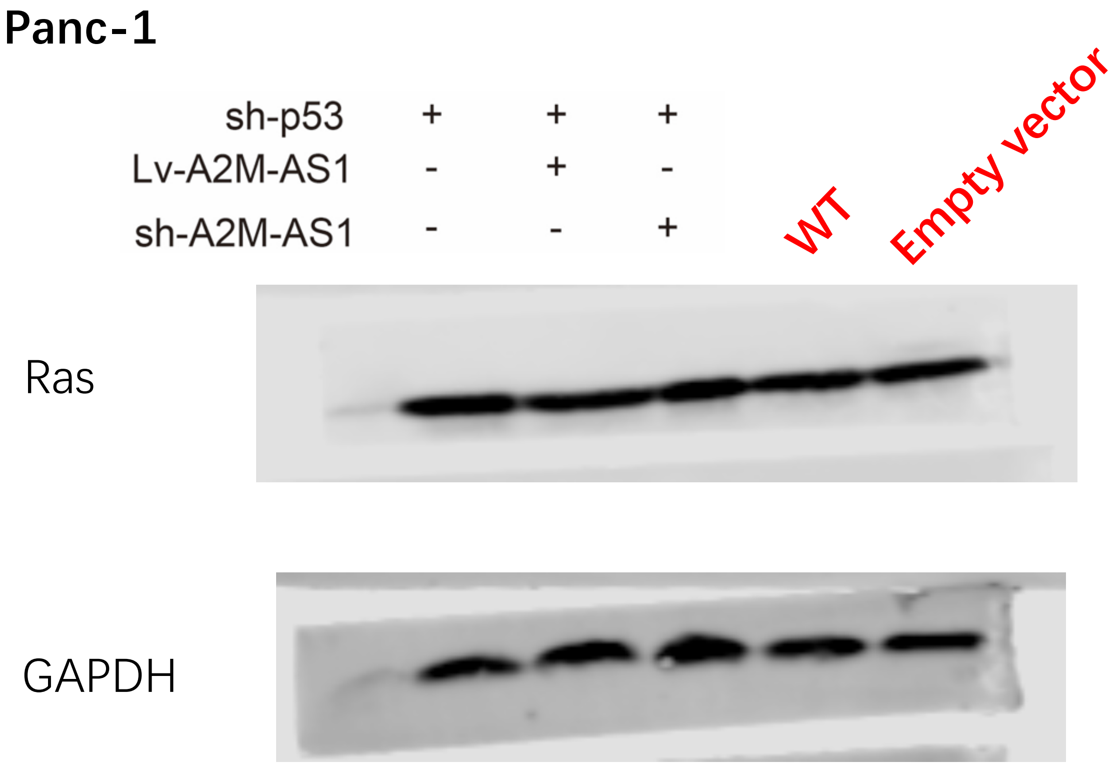

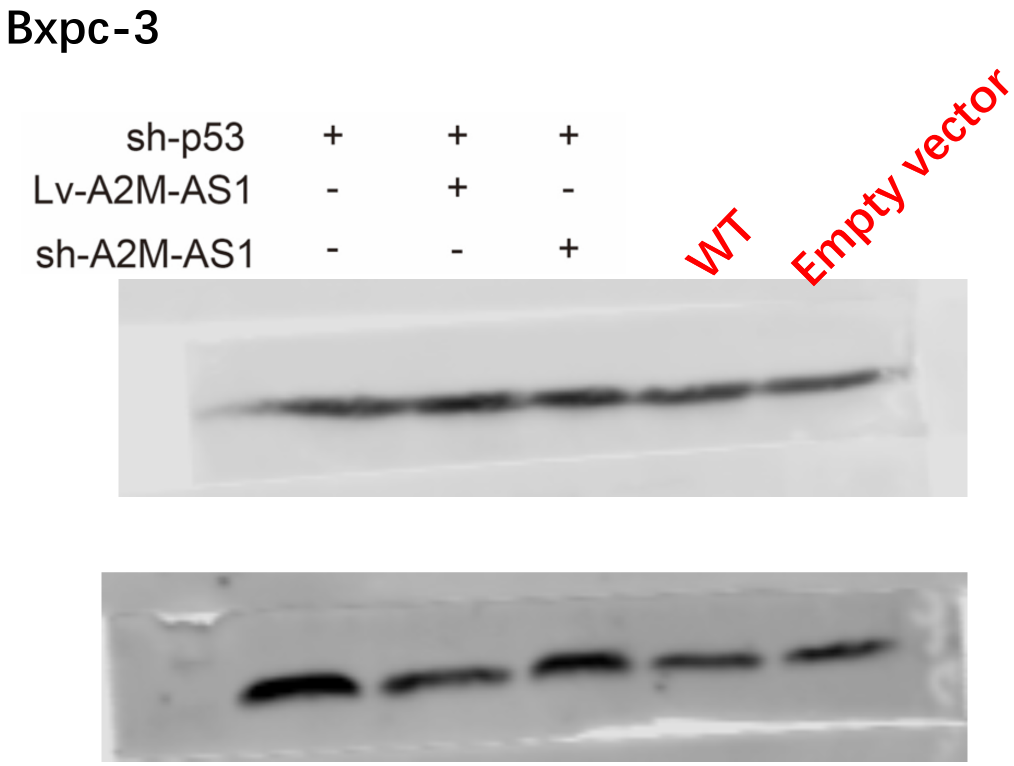

Supplement: Supplementary file 1 — Data S1. [file CAM4-14-e70956-s002.pdf]
