# Supplementary material for: Knockdown of p53 Enhances LncRNA A2M‐AS1 Inhibition of Pancreatic Cancer Progression via Regulating MAPK Pathway
Source: Cancer Med. 2025 Jul 13;14(13):e70956. doi: 10.1002/cam4.70956 (PMC12256274; doi:10.1002/cam4.70956)
Supplement: Supplementary file 2 — Table S1. Clinical baseline data of pancreatic cancer patients and the correlation between the expression levels of p53 and A2M‐AS1 and clinical characteristics of pancreatic cancer patients. [file CAM4-14-e70956-s001.docx]

**Table S1** Clinical baseline data of pancreatic cancer patients and the correlation between the expression levels of p53 and A2M-AS1 and clinical characteristics of pancreatic cancer patients

|  |  | **p53 level** | | |  | **A2M-AS1 level** | | |  |
| --- | --- | --- | --- | --- | --- | --- | --- | --- | --- |
| **Factor** |  | **Total** | **Low** | **High** | ***P*-value** | **Total** | **Low** | **High** | ***P*-value** |
| **Age** |  |  |  |  |  |  |  |  |  |
| ＜60y |  | 16 | 3 | 13 | 0.626 | 16 | 14 | 2 | 1 |
| ≥60y |  | 16 | 2 | 14 |  | 16 | 14 | 2 |  |
| **Sex** |  |  |  |  |  |  |  |  |  |
| Male |  | 19 | 2 | 17 | 0.337 | 19 | 18 | 1 | 0.135 |
| Female |  | 13 | 3 | 10 |  | 13 | 10 | 3 |  |
| **Smoke** |  |  |  |  |  |  |  |  |  |
| yes |  | 10 | 2 | 8 | 0.646 | 10 | 9 | 1 | 0.773 |
| no |  | 22 | 3 | 19 |  | 22 | 19 | 3 |  |
| **drink** |  |  |  |  |  |  |  |  |  |
| yes |  | 9 | 2 | 7 | 0.52 | 9 | 8 | 1 | 0.082 |
| no |  | 23 | 3 | 20 |  | 23 | 20 | 3 |  |
| **T stage** |  |  |  |  |  |  |  |  |  |
| T1/T2 |  | 20 | 4 | 16 | 0.379 | 20 | 17 | 3 | 0.581 |
| T3/T4 |  | 12 | 1 | 11 |  | 12 | 11 | 1 |  |
| **N stage** |  |  |  |  |  |  |  |  |  |
| N0 |  | 17 | 5 | 12 | 0.022 | 17 | 15 | 2 | 0.893 |
| N1/N2 |  | 15 | 0 | 15 |  | 15 | 13 | 2 |  |
| **Pathology**  **grade** |  |  |  |  |  |  |  |  |  |
| I |  | 11 | 4 | 7 | 0.132 | 11 | 10 | 1 | 0.805 |
| II |  | 15 | 1 | 14 |  | 15 | 13 | 2 |  |
| III |  | 2 | 0 | 2 |  | 2 | 2 | 0 |  |
| IV |  | 4 | 0 | 4 |  | 4 | 3 | 1 |  |
| **Differentiated degree** |  |  |  |  |  |  |  |  |  |
| M/L |  | 29 | 5 | 24 | 0.434 | 29 | 25 | 4 | 0.492 |
| H |  | 3 | 0 | 3 |  | 3 | 3 | 0 |  |
| **Distant metastasis** |  |  |  |  |  |  |  |  |  |
| yes |  | 4 | 0 | 4 | 0.358 | 4 | 3 | 1 | 0.419 |
| no |  | 28 | 5 | 23 |  | 28 | 25 | 3 |  |
| **Perineural invasion** |  |  |  |  |  |  |  |  |  |
| yes |  | 29 | 5 | 24 | 0.434 | 29 | 25 | 4 | 0.492 |
| no |  | 3 | 0 | 3 |  | 3 | 3 | 0 |  |
| **Vascular cancer thrombus** |  |  |  |  |  |  |  |  |  |
| yes |  | 13 | 1 | 12 | 0.307 | 13 | 12 | 1 | 0.492 |
| no |  | 19 | 4 | 15 |  | 19 | 16 | 3 |  |
| **Tumor location** |  |  |  |  |  |  |  |  |  |
| head |  | 24 | 3 | 21 | 0.399 | 24 | 20 | 4 | 0.217 |
| Body/tail |  | 8 | 2 | 6 |  | 8 | 8 | 0 |  |
